# Spherulite-like Ni(II)(l-glutaminato·H_2_O)_2_ Complex: Morphological Microstructure, DFT-Assisted Crystal Structure Determination, and Vibrational Analysis

**DOI:** 10.1021/acsomega.4c10609

**Published:** 2025-03-02

**Authors:** Wesley K. C. Oliveira, Raisa M. C. S. Diniz, Jéssica A. O. Rodrigues, Francisco F. de Sousa, Clenilton C. dos Santos, Francisco S. M. Sinfrônio, Fabio F. Ferreira, José G. da Silva Filho, Alan S. de Menezes

**Affiliations:** † Department of Physics, CCET, Federal University of Maranhão - UFMA, 65080-805, São Luís, MA, Brazil; ‡ Center for Social Sciences, Health, and Technology, Federal University of Maranhão - UFMA, 65900-410, Imperatriz, MA, Brazil; § Department of Mathematics and Informatics, State University of Maranhão - UEMA, 65055-310, São Luís, MA, Brazil; ∥ Department of Electrical Engineering, Federal University of Maranhão, CCET, 65080-805, São Luís, MA, Brazil; ⊥ Institute of Chemistry, Federal University of Rio Grande do Norte - UFRN, 59078-970, Natal, RN, Brazil; # Center for Natural and Human Sciences, Federal University of ABC (UFABC), 09210-580, Santo Andre, SP, Brazil; ∇ Faculdade de Educação, Ciências e Letras do Sertão Central, Universidade Estadual do Ceará - UECE, 63902-098, Quixada, CE, Brazil; ○ Institute of Exact and Natural Sciences, Federal University of Para - UFPA, 66075-110, Belem, PA, Brazil

## Abstract

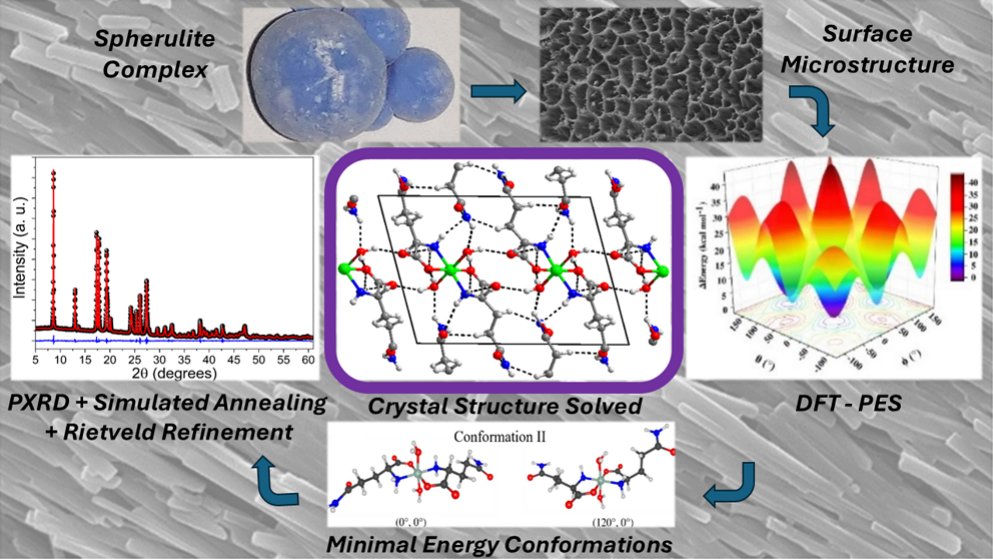

l-glutamine complexes with transition metals have already been reported in the literature, but there have been no studies on l-glutamine complexes with Ni^2+^. Here, the new polycrystalline spherulite-like Ni(II)(l-glutaminato·H_2_O)_2_ complex was synthesized, and powder X-ray diffraction (PXRD) measurements and density functional theory (DFT) calculations were performed to determine its crystal structure. SEM images revealed the beautiful morphological microstructure of the new spherulite-like complex’s surface and internal region. Thermogravimetric analysis (TGA) and vibrational spectroscopic techniques (infrared and Raman) were also performed. A mass loss observed at around 160 °C, equivalent to the mass of two water molecules, was determined by TGA. DFT calculations were initially performed to generate the most stable molecule and assist in crystal structure determination using the PXRD data. Five conformations of the complex were established, and these structures were optimized with the B3LYP hybrid functional and Def2-tzvp basis function for the nickel atom and 6-311++G(d,p) for the other atoms. Scanning calculations were performed to construct the potential energy surfaces and identify the most stable structure. From a PXRD measurement and the possible configurations of the optimized molecules, the Ni(II)(l-glutaminato·H_2_O)_2_ crystal structure was solved using a simulated annealing approach. The new compound crystallized in a triclinic system with a *P*1̅ space group.

## 1 Introduction

Transition metal complexes with amino acids play crucial roles in oxygen transport within the blood, photosynthesis, biological and industrial catalysis, and treating diverse pathological conditions. Furthermore, they can exhibit important physical properties such as electrical,^1^ magnetic,^2^ and nonlinear optics.^3^ Therefore, synthesizing and investigating the structure of these new complexes aid in comprehending metal–ligand interactions and their physical properties.

Amino acids can easily complex with transition metal and rare earth ions acting as monodentate ligands, usually in acidic pH solution, like for Cu(II)(β-alanine)Cl_2_,^4^ as bidentate ligands from neutral to alkaline pH solution; this is the case for most of the complexes with amino acids and as tridentate ligands depending on the amino acid side chain, like for Ni(II)(l-histidinato)_2_·H_2_O.^5^ Those transition metal complexes can grow in the anhydrous form, without water molecules in the crystal structure,^6,7^ and in the hydrated form, with one or more water molecules bonded by hydrogen bonds (hydration water),^5^ with one^8,9^ or two water molecules^10^ coordinated to the metal ion (coordinated water), or with both kinds of water molecules.^11,12^

The spatial way in which amino acids bind to the metal ion can assume the *cis*(13,14) and *trans*(10) configurations and also the way presented in the diaqua-bis(l-valinato)-nickel(ii) crystal^15,16^ and Ni(II)(l-threoninato)_2_·H_2_O.^17^ A study was also carried out on the *cis–trans* isomerism of the bis(l-valinato)copper(II) molecule due to hydration. The thermogravimetric curves showed two mass loss events for the hydrated crystal: the first one, from 30 to 220 °C caused by the water molecules release, and the second one, from 190 to 270 °C, caused by sample partial sublimation and decomposition.^14^ The stability study of the [bis(l-alaninato)diaqua]nickel(II) dihydrate crystal using Raman scattering, powder X-ray diffraction (PXRD), as a function of the temperature, and thermal analysis were reported by Baldez et al.^12^ PXRD showed that the crystal undergoes two phase transformations at high temperatures due to the loss of water molecules. A vibrational study of these complexes using FT-IR and Raman spectroscopy combined with DFT calculations can provide the complete assignment of the vibrational modes of these complexes, like the studies performed for the bis(β-alanine)nickel(II) dihydrate crystal.^18^

In most cases, it is possible to obtain those complexes growing single crystals of enough size to perform X-ray diffraction measurements in a single crystal diffractometer to determine their crystal structure. However, some materials can only be obtained as polycrystals. In those cases, different methods/algorithms can be employed to determine structural parameters like unit cell parameters, space group, and atomic positions.^19^ Simulated Annealing^20^ and Parallel Tempering^21^ algorithms, as global optimization methods for structure determination^19^ based on the Reverse Monte Carlo model,^22^ are highlighted as important methods. The best model depends on the complexity of the structure and the instrumental conditions. Direct-space methods can be used to determine the crystal structure by using PXRD data. In parallel, ab initio quantum mechanical calculations based on density functional theory (DFT) can be used to calculate the minimum energy structure for these molecules, assisting in structure determination or directly comparing theory and experiment.

Performing a search in the Cambridge Crystallographic Data Center (CCDC) database, only two complexes of Cu(II) and Zn(II) with glutamine were found; the complex with Cu(II) is monoclinic with space group *C*_2_(23,24) and the one with Zn(II) is monoclinic with space group *P*2_1_.^25^ We could not find any nickel-containing glutamine complex. Spherulites are radially polycrystalline aggregates with an outer spherical shape that polymers, minerals, inorganic crystals, metals,^26^ organic small molecules like tryptophan^27^ and curcumin,^28^ and large organic molecules^29^ can form. Searching in the literature, we could not find examples of complexes grown as spherulites. To the best of our knowledge, this is the first report of a spherulite-like complex material.

Therefore, the present work reports the synthesis, morphology, and crystal structure determination of the novel spherulite-like Ni(II)(l-glutaminato·H_2_O)_2_ complex through powder X-ray diffraction data, using a simulated annealing approach, and confirms its validation by Rietveld refinement. We also used DFT calculations to assist the structure determination from PXRD data, providing the initial optimized complex molecule necessary for the structure determination. In addition, SEM/EDS measurements, thermal studies using thermogravimetric analysis (TGA), and vibrational studies using infrared (IR) and Raman spectroscopy techniques were also performed, and DFT was also used for the complete assignment of the vibrational modes of this new complex.

## 2 Results and Discussion

### 2.1 Initial Sample Characterization

**Figure 1:**
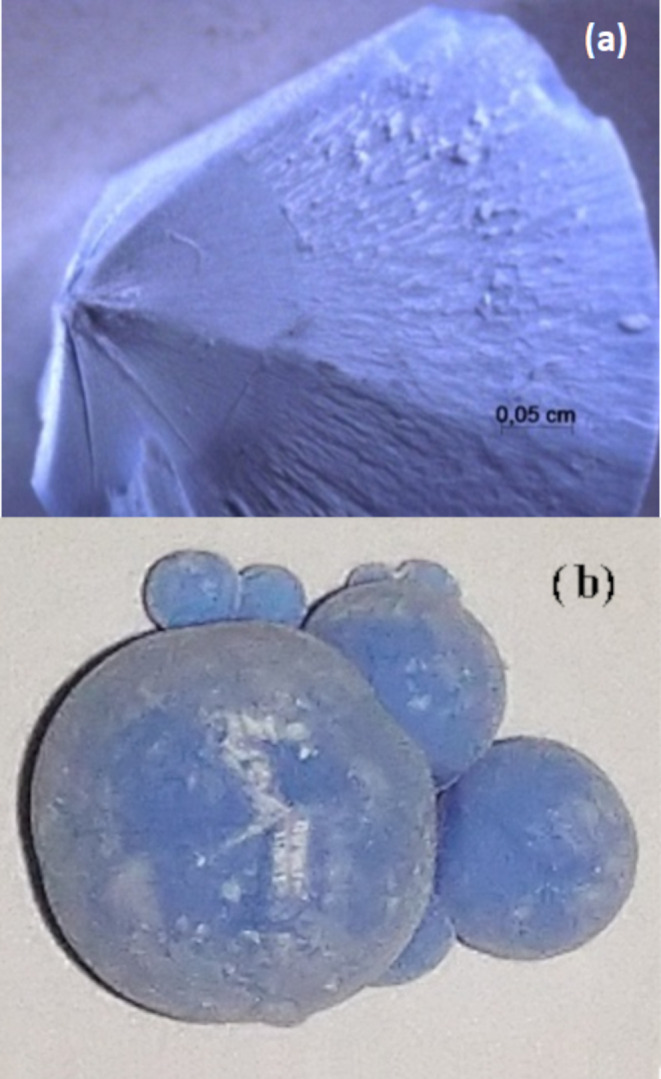
(a) Optical microscopy image of the internal region of the spheres and (b) the photograph of the spheres showing its top surface.

The new nickel-containing l-glutamine complex, obtained by the slow evaporation method, crystallized in blue polycrystalline spheres. The internal part of the spheres and the external morphology can be seen in Figure 1a,b. It is possible to see that polycrystalline spheres have spherulite-type growth.

**Figure 2:**
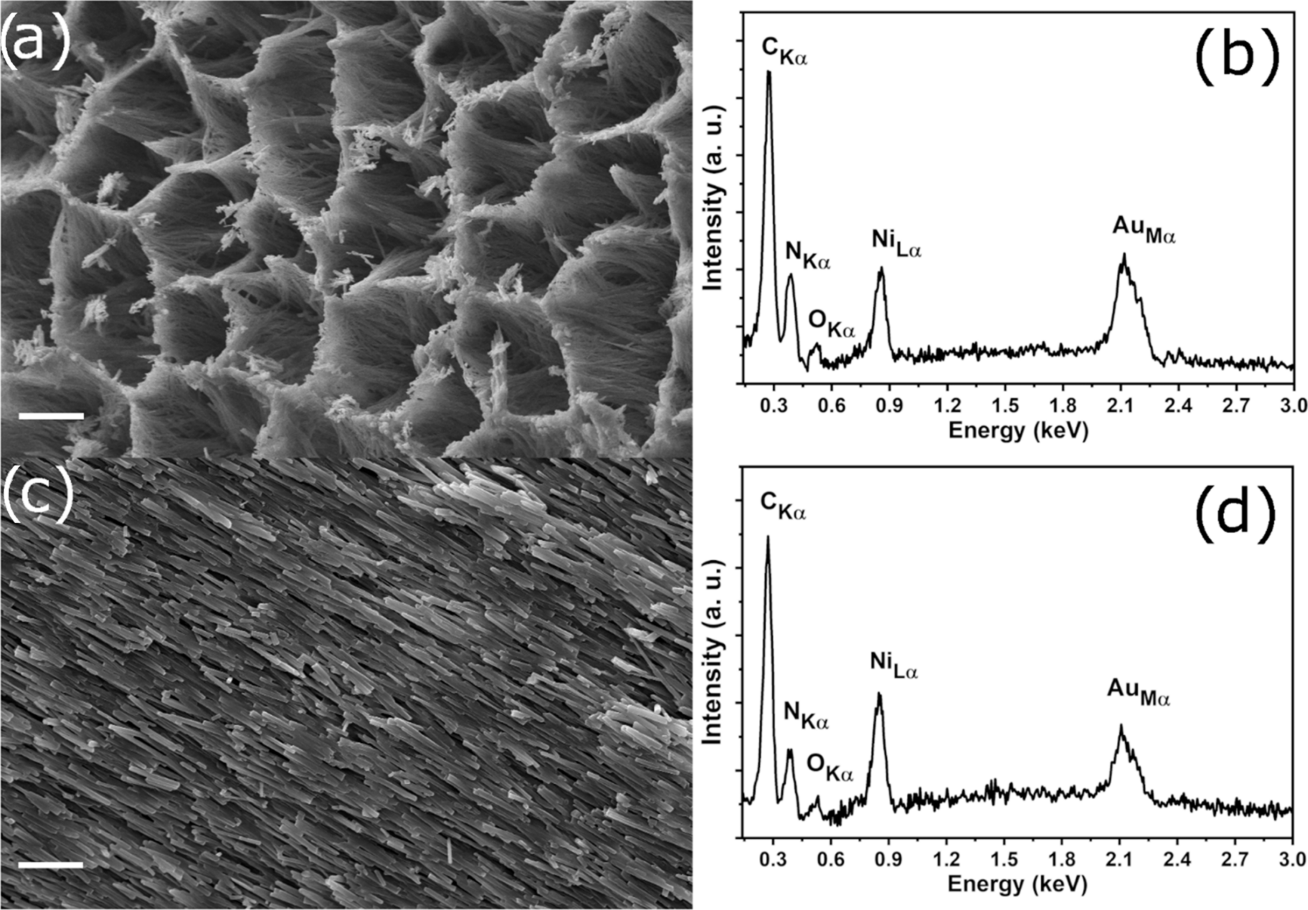
(a) SEM image and (b) EDS of the top surface of the sphere; (c) SEM image and (d) EDS of the internal region of the spheres. SEM measurements conditions: high vacuum, SE detector, EHT = 15 kV, WD = 8.5, I_probe_ = 12 nA, (a) Mag = 2 kX, scale bar = 10 μm, and (b) Mag = 4 kX, scale bar = 5 μm. Au signal comes from the metallization process.

SEM and EDS measurements were performed to characterize the surface and the internal region of the spheres in detail. Figure 2a shows the SEM of the sphere’s surface, where one can see several structures (like irregular honeycombs) formed by microrods. The EDS spectra (Figure 2b) show the presence of C, N, O, and Ni atoms, evidencing the purity of the sample. Figure 2c shows the sphere’s internal region, where one can see well-defined and aligned microrods with approximately 400 nm thickness and length varying from 1 to 8 μm. More images of the beautiful and amazing microstructure of this new spherulite-like l-glutamine complex can be seen in Figures S1 and S2 of the Supporting Information file.

**Figure 3:**
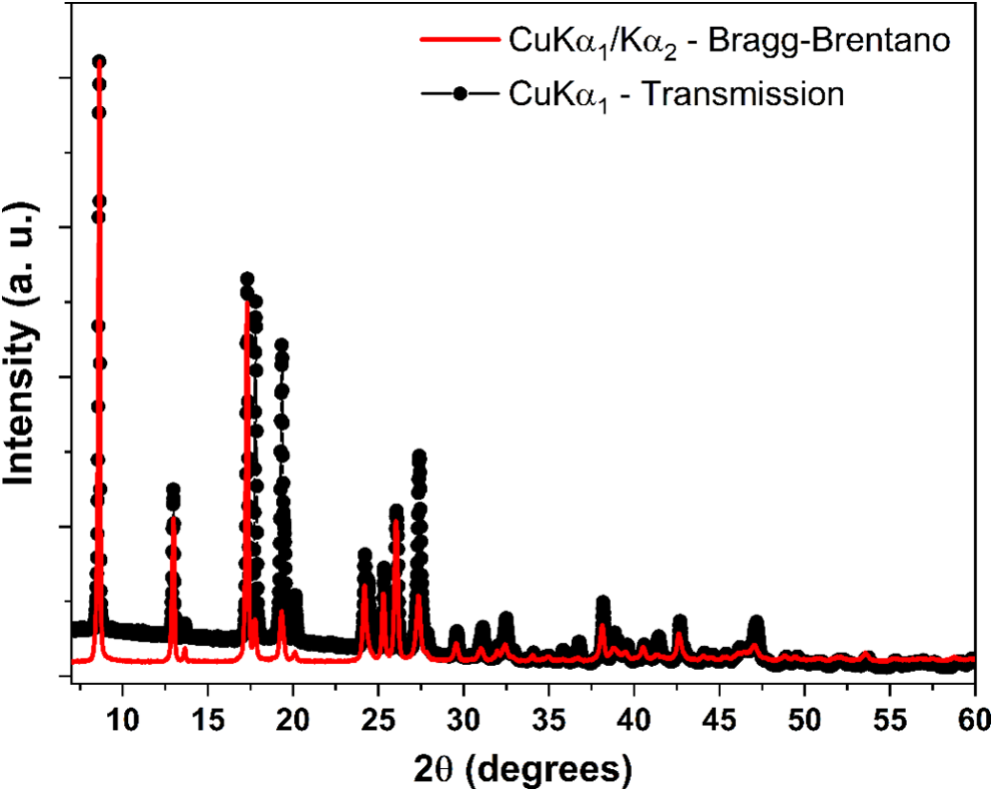
PXRD patterns in the Bragg–Brentano geometry and transmission geometry.

The spheres were transformed to powder to perform powder X-ray diffraction (PXRD) measurements to identify the complex’s crystal structure. Figure 3 shows the PXRD of the complex performed in a diffractometer with Cu*K*α_1_/*K*α_2_ radiation in the Bragg–Brentano geometry and performed in a diffractometer with monochromatic Cu*K*α_1_ radiation in the transmission geometry. Comparing both patterns, one can see a difference in the relative intensity because measurements in Bragg–Brentano geometry are more susceptible to preferred orientation effects.

**Figure 4:**
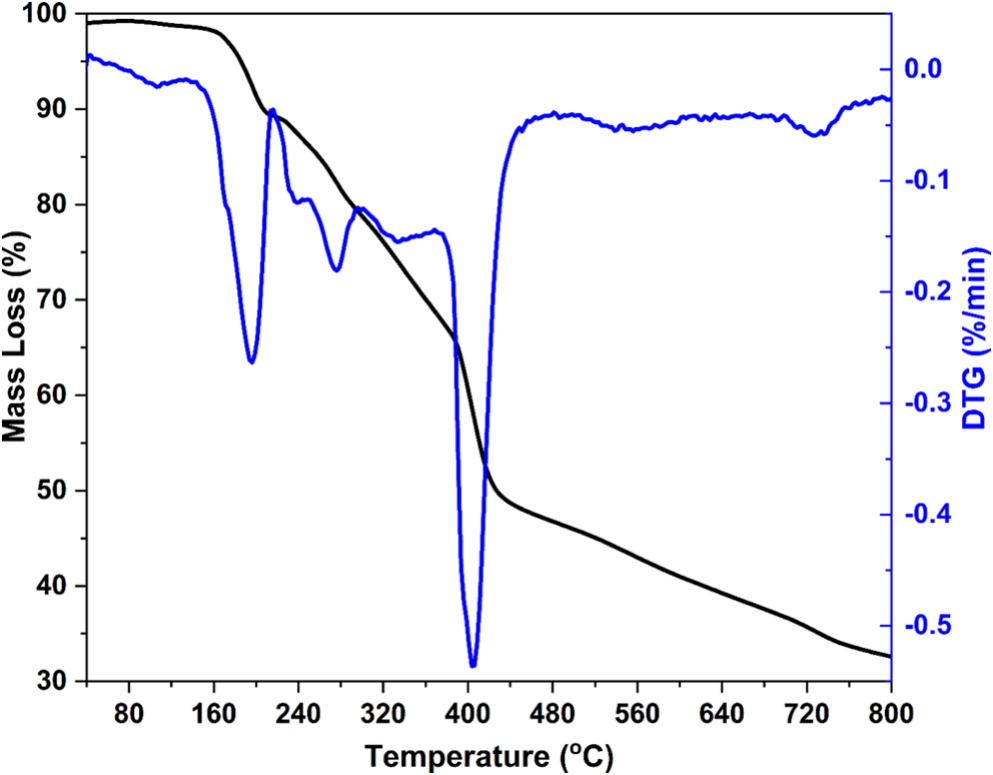
TGA and DTG curves of the Ni-containing l-glutamine complex showing the mass losses due to water molecule release and l-glutamine decomposition.

After performing a search in the Cambridge Crystallographic Data Center (CCDC), no X-ray diffraction pattern matching this one was found. Therefore, one can conclude that this is a new material. Prior knowledge of the chemical structure of a new material can help to determine its structure. As crystals of amino acid complexes with transition metals can grow with water molecules in their crystal structure, thermogravimetry analysis (TGA) measurement was carried out to detect any mass loss associated with water molecules release, as seen in Figure 4. A first mass loss of around 160 °C of about 9% corresponds to the mass of two water molecules. The other mass losses can be attributed to decomposition of the l-glutamine molecule.

The release of water molecules at elevated temperatures suggests that the two molecules are coordinated to the Ni^2+^ ion, as water molecules bound only by hydrogen bonds are usually released at close to 100 °C. Consequently, the chemical formula of this new complex is proposed as Ni(II)(C_5_H_9_N_2_O_3_·H_2_O)_2_ and was considered in the determination of its crystal structure.

### 2.2 DFT Structural Analysis

**Figure 5:**
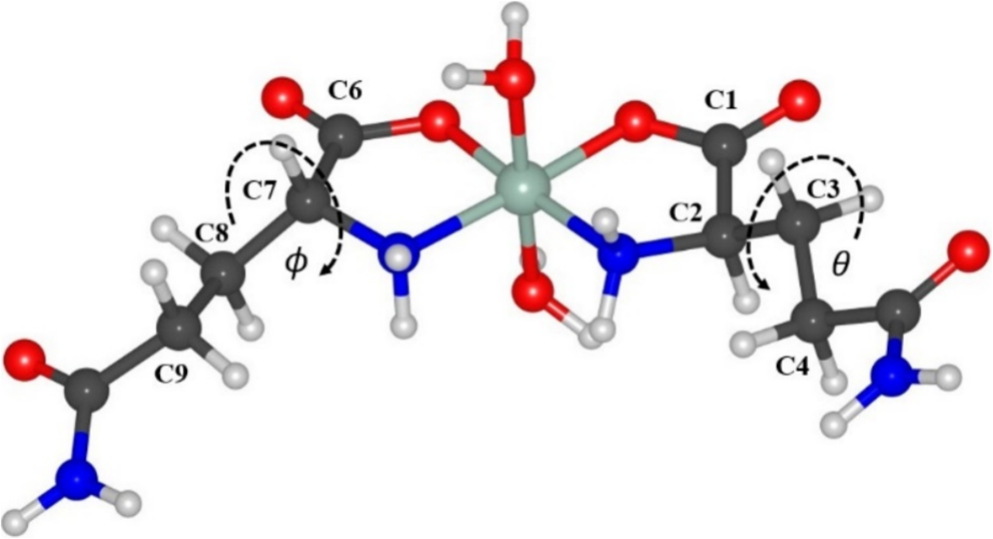
Dihedral angles θ and ϕ C1–C2–C3-C4 and C6–C7–C8-C9, respectively.

To evaluate the lowest energy geometry of the molecular complex, five different conformations were proposed for the Ni(II)(l-glutaminato·H_2_O)_2_ molecule according to the relative orientation of the amino acid, as shown in Figure S3 of the Supporting Information file. After geometry optimization of those structures, an energy scan was performed concerning the dihedral angles theta (θ) and phi (ϕ), as shown in Figure 5. Table S1 (see the Supporting Information) presents the geometric parameters, including bond lengths and dihedral angles of the lowest-energy structures obtained from the scan process for the five conformations of the complexes.

**Figure 6:**
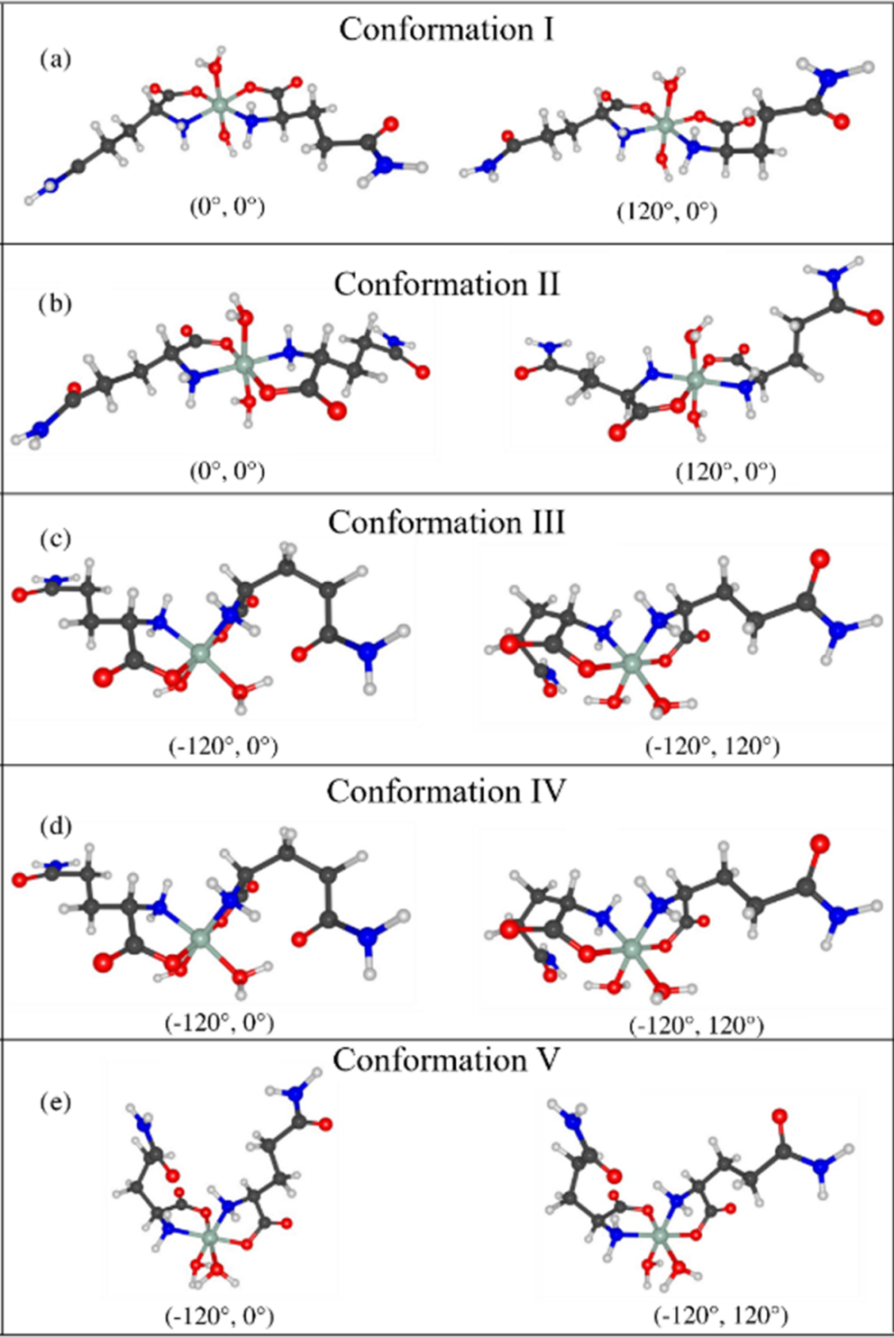
Two lowest-energy structures for each set of dihedral angles θ and ϕ for the conformations (a) I, (b) II, (c) III, (d) IV, and (e) V.

The potential energy surface of each conformation is shown in Figure S4 of the Supporting Information file. Each surface exhibits a global energy minimum point, identifying the most stable structure. However, conformations with formation energy within the range of thermal energy at room temperature (*k*_B_*T* ≈ 0.59 kcal mol^–1^) were also considered in our analysis. The formation energy^30,31^ was calculated to evaluate the stability of the minimized low-energy structures of each conformation, following the equation
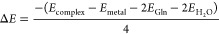
1where *E*_complex_ is the energy of the complex being studied (I, II, III, IV, or V), *E*_metal_ is the energy of the nickel atom, 2*E*_Gln_ is the energy of the two glutamine molecules in the complex, and 2*E*_H2O_ is the energy of the two water molecules. These energies were obtained from single-point calculations combined with vibrational frequency analyses of the complex and the individual ligands in the gas phase.^32^ For complexes I and II, the (0°, 0°) dihedral set presents the most stable structure possible, with energy differences from the (120°, 0°) dihedrals of 0.07 and 0.38 kcal/mol, respectively. The remaining complexes show energy variations above 0.59 kcal mol^–1^. Even considering thermal effects, these local minima would still exhibit instability at room temperature. Figure 6 shows the two lowest-energy structures obtained for each of the analyzed conformation.

Given the relatively small energy difference between the two lowest-energy configurations for complexes I and II, it is likely that both configurations could be experimentally observed. However, subsequent analyses will focus exclusively on the global minima of each conformation, as these represent the most thermodynamically favorable structures and are the most relevant for understanding the complexes’ behavior under standard experimental conditions.

Using the global minimum data of each structure, it was possible to evaluate the thermodynamic functions under standard conditions, including inner energy (Δ*U*), enthalpy (Δ*H*), and Gibbs free energy (Δ*G*) for the five conformations under study, as presented in Table 1.

**Table 1:** Comparison of the Inner Energy (ΔU), Enthalpy (Δ*H*), and Gibbs Energy (Δ*G*) of the Five Conformations Expressed in kcal·mol^–1^

conformation	Δ*U* (kcal·mol^–1^)	Δ*H* (kcal·mol^–1^)	Δ*G* (kcal·mol^–1^)
I	–113.579	–112.980	–122.364
II	–162.525	–161.926	–171.310
III	–109.814	–110.361	–120.482
IV	–111.069	–109.477	–119.227
V	–110.442	–110.267	–119.854

As one can see in Table 1, conformation II with (θ, ϕ) = (0°, 0°) displays the lowest values for all thermodynamic functions, with Δ*U* = −162.525 kcal/mol, Δ*H* = −161.926 kcal·mol^–1^, and Δ*G* = −171.310 kcal·mol^–1^, indicating it as the most thermodynamically favorable structure. Consequently, this conformation was selected as the basis for determining the crystal structure of Ni(II)(l-glutaminato·H_2_O)_2_, which was used for further analyses.

### 2.3 Crystal Structure Determination

All steps in determining the crystal structure, including indexing, Pawley refinement, simulated annealing, and Rietveld refinement, were carried out using the TOPAS-academic V7 program. The first 23 peaks were used in the indexing step, and the best solutions were obtained for the triclinic system with the *P*1̅ space group. After Pawley refinement, the following lattice parameters were obtained: *a* = 5.1369(2)Å, *b* = 10.6011(8)Å, *c* = 14.1979(8)Å, α = 104.402(7)°, β = 96.613(4)°, and γ = 91.308(8)°. The Pawley refinement graph can be seen in Figure S5 of the Supporting Information file.

In the next step, the Simulated Annealing method was used to determine the atomic positions of the atoms in the unit cell. The rigid body of the ten conformations presented in Figure 6, written in Z-matrix format, was inserted into a TOPAS input file (INP file) for each molecular conformation. All ten conformations were submitted to the simulated annealing step. There was no convergence of *R*_wp_ values (>20%) for the molecules III, IV, and V. However, conformation I (*cis*) and II (*trans*) showed *R*_wp_ values of 14.57 and 13.14%, respectively. The structures obtained with the solutions (0°, 0°) and (120°, 0°) did not show significant differences in *R*_wp_ values for both conformations I and II. Therefore, we decided to consider the crystalline structure obtained with the (0°, 0°) solution (conformation II) as the crystalline structure of the new Ni(II)(l-glutaminato·H_2_O)_2_ complex.

**Figure 7:**
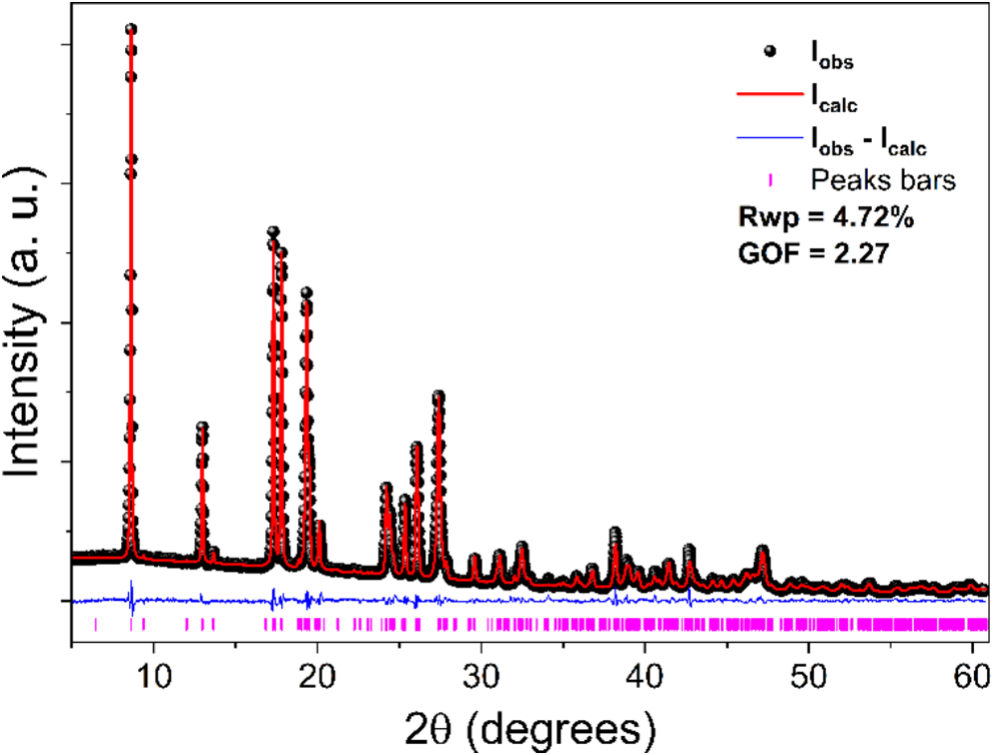
Final Rietveld refinement of the Ni(II)(l-glutaminato·H_2_O)_2_.

The obtained structure was refined using the Rietveld method, and its graph is shown in Figure 7. Due to the difference between these patterns and the *R*_wp_ and GOF values, an excellent fit between the calculated and experimental PXRD patterns can be observed.

Table 2 presents crystallographic data obtained after final Rietveld crystal structure refinement. The Supporting Information presents other crystallographic information such as atomic positions (Table S2), lengths of the bonds (Table S3), bond angles (Table S4), and hydrogen bond lengths and angles (Table S5).

**Table 2:** Crystallographic Data of the Ni(II)(l-glutaminato·H_2_O)_2_ Crystal

chemical formula	C_10_H_22_N_4_NiO_8_
molecular weight (g mol^–1^)	385.01
crystal system	triclinic
space group	*P*1̅
*a* (Å)	5.1308(2)
*b* (Å)	10.6038(9)
*c* (Å)	14.2084(14)
α (deg)	104.325(8)
β (deg)	96.652(4)
γ (deg)	91.433(8)
volume (Å^3^)	742.79(11)
*Z*	2
density (g cm^–3^)	1.7214
absorption coefficient (mm^–1^)	2.366
F(0,0,0)	404
data collection (2θ, °)	5.0 to 60.135 (resolution: 1.513 Å)
range of indices *h*, *k*, *l*	0 ≤ *h* ≤ 3, – 6 ≤ *k* ≤ 7, – 9 ≤ *l* ≤ 9
total number of reflections	455

**Figure 8:**
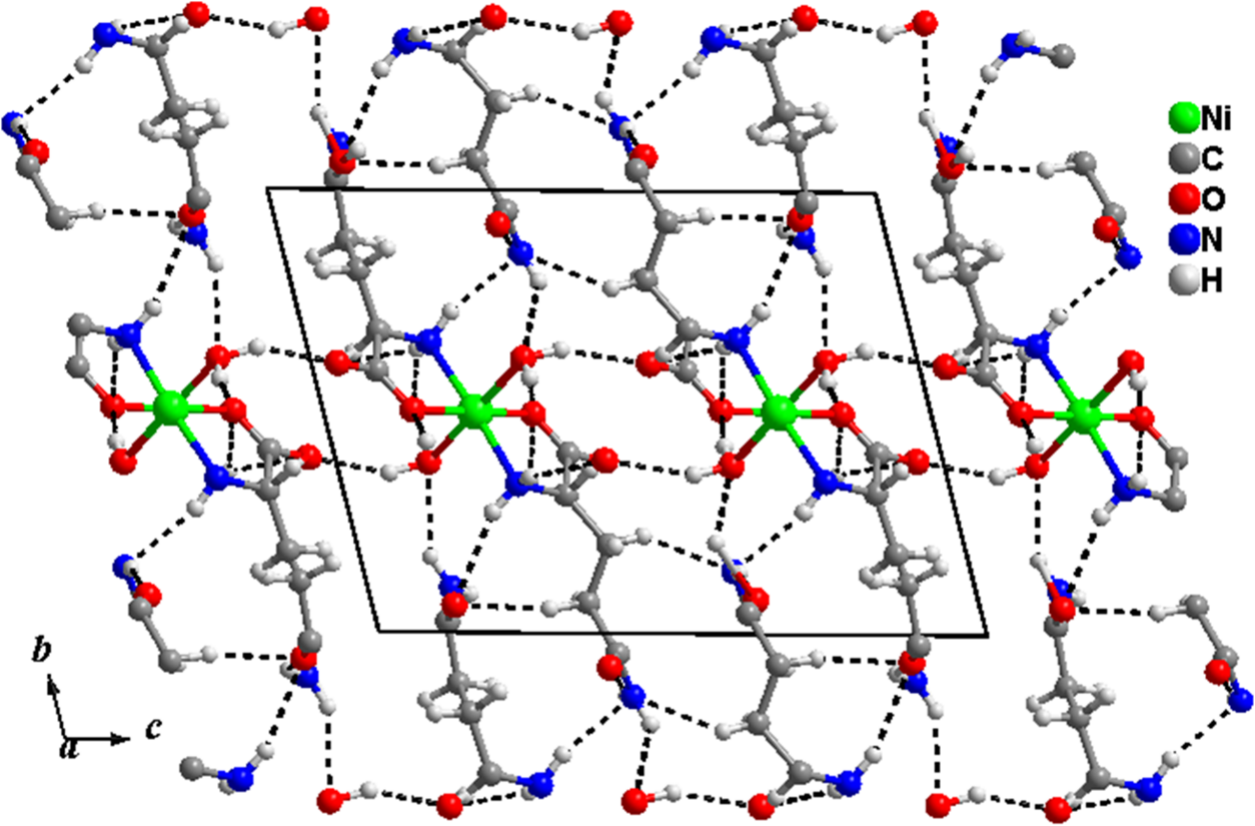
Unit cell, along the *a*-axis, of the Ni(II)(l-glutaminato·H_2_O)_2_ crystal showing the hydrogen bonds (dashed lines).

The crystal unit cell was generated with the final data obtained from a Rietveld refinement, such as lattice parameters and atomic positions. Figure 8 shows the unit cell along the *a*-axis, with hydrogen bonds (dashed lines).

**Figure 9:**
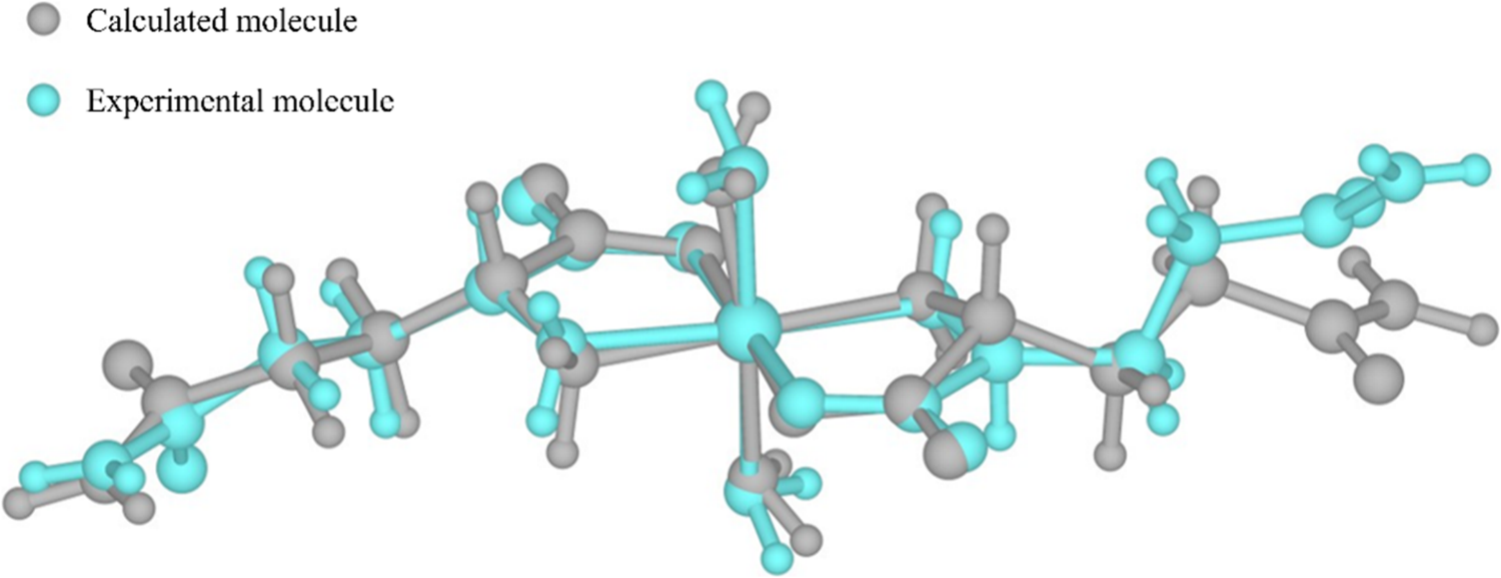
Superposition of Ni(II)(l-glutaminato·H_2_O)_2_ molecules calculated by DFT for conformation II and experimental molecule.

Figure 9 shows the overlap of the molecules calculated by DFT for conformation II and the experimental. In this overlap, a good match is observed in most of the two molecules, except at the end of one of the l-glutamine molecules. This shows that the DFT calculations generated an excellent starting point for resolving the crystal structure using powder X-ray diffraction.

### 2.4 Vibrational Analysis

The vibrational analysis of the complex was carried out considering the most stable conformational structure previously determined (conformation II). Infrared (IR) and Raman spectroscopy determined the presence of important functional groups in the Ni(II)(l-glutaminato·H_2_O)_2_ crystal. According to the PXRD analysis, in this conformation, the Ni(II)(l-glutaminato·H_2_O)_2_ crystal has a triclinic *P*1̅ (*C*_*i*_^1^) structure (*Z* = 2), wherein all of the atoms are occupying *C*_1_ (2*i*) symmetry sites. Consequently, the distribution of the vibrational modes of Ni(II)(l-glutaminato·H_2_O)_2_ in terms of the irreducible representation of the factor group *C*_*i*_ is Γ = 135A_g_ + 135A_u_, where 3A_u_ are acoustic modes, and 135A_g_ + 132A_u_ are optical modes. The 135A_g_ modes are Raman-active modes, while the 132A_u_ modes are IR-active modes.

**Figure 10:**
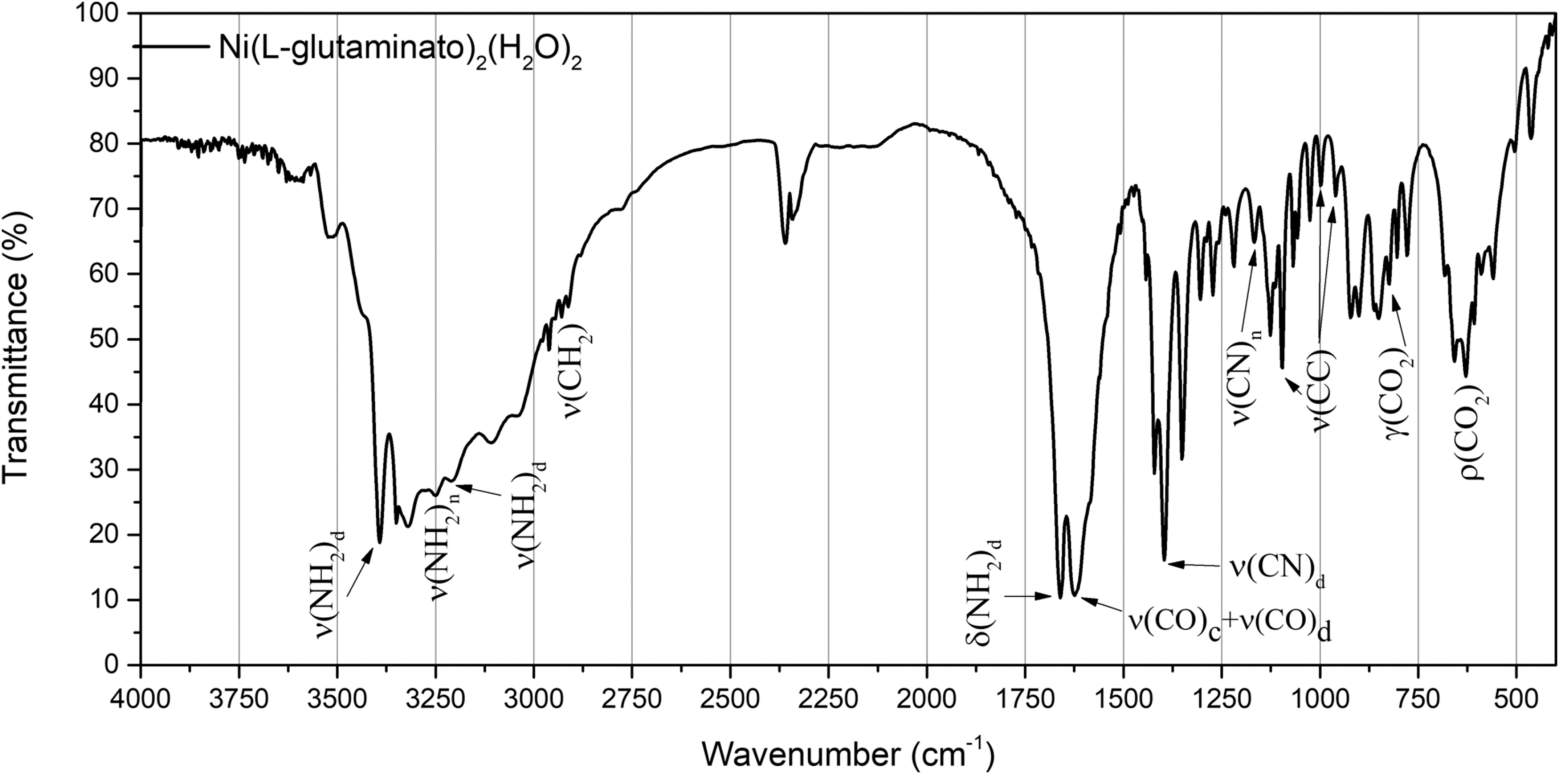
IR spectrum of the Ni(II)(l-glutaminato·H_2_O)_2_ crystal in the 4000–400 cm^–1^ range.

The IR spectrum of the complex can be seen in Figure 10. The assignments and comparisons of the main observed bands with values found in the literature are summarized in Table 3.

**Table 3:** Infrared Modes of the Ni(II)(l-glutaminato·H_2_O)_2_ Crystal Compared to Assignments Reported in the Literature for Similar Systems^a^

crystal (cm^–1^)	literature (cm^–1^)	assignment	ref
3523	3560	ν(OH)	(33)
3392	3381	ν(NH_2_)_d_	(34)
3247	3265	ν(NH_2_)_n_	(34)
3201	3186	ν(NH_2_)_d_	(34)
2929	2927	ν(CH_2_)	(34,35)
1660	1680	δ(NH_2_)_d_	(34)
1623	1637	ν(CO)_c_ + ν(CO)_d_	(34,35)
1580	1585	δ(NH_2_)_n_	(34)
1420	1414	δ (CH_2_)	(34)
1396	1363	ν(CN)_d_	(34,35)
1303	1308	δ(CH_2_) + ν(C–CO_2_)	(34)
1255	1255	τ(CH_2_) + ρ(CH_2_)	(34)
1218	1225	τ(CH_2_)	(34)
1166	1157	ν(CN)_n_	(34)
1095	1117	ν(CC)	(34)
997	1022	ν(CC)	(34)
960	960	ν(CC)	(34,35)
823	872	γ(CO_2_)	(34,35)
779	783	γ(NH_2_)_n_	(34)
660	669	γ(NH_2_)_d_	(34)
628	607	ρ(CO_2_)	(34)

a ν - stretching, δ - angular deformation, τ - twist, γ - out-of-plane deformation, ρ - rotation.

In our study, the band at 3523 cm^–1^ is associated with the stretching of the OH group belonging to the water molecules present in the complex. According to Figure 10, the vibrations related to the stretching of the NH bond, ν(NH_2_)_d_, are observed at 3392 and 3201 cm^–1^ for the amide group and at 3247 cm^–1^ for the amine group, ν(NH_2_)_n_. The peak centered at around 2929 cm^–1^ refers to the ν(CH_2_) bond stretching.

The mode associated with 1660 cm^–1^ was designated as an angular deformation of the amide group, δ(NH_2_)_d_, while the peak at 1580 cm^–1^ corresponds to the angular deformation of the amine group δ(NH_2_)_n_. The combination of stretch-type movements ν(CO)_c_ + ν(CO)_d_ of the carboxylic and amide groups was identified at 1623 cm^–1^. The peak around 1420 cm^–1^ was associated with an angular deformation δ(CH_2_). The CN bond stretching of the amide group, ν(CN)_d_, was observed at around 1396 cm^–1^. The peaks located at 1303 and 1255 cm^–1^ correspond to the combination of movement types δ(CH_2_) + ν(C–CO_2_) and τ(CH_2_) + ρ(CH_2_), respectively. The peak at 1218 cm^–1^ corresponds to the τ(CH_2_).

The band observed at 1166 cm^–1^ corresponds to ν(CN_2_)_n_ bond stretching of the amine group. The CC bond stretch, ν(CC), was identified at 1095, 997, and 960 cm^–1^. The vibrations related to out-of-plane deformations and rotations of CO_2_, such as γ(CO_2_) and ρ(CO_2_)_d_, were found in 823 and 628 cm^–1^, respectively. The peaks at 779 and 660 cm^–1^ were associated with out-of-plane deformations of the amine group, γ(NH_2_)_n_, and the amide group, γ(NH_2_)_d_, respectively.

**Figure 11:**
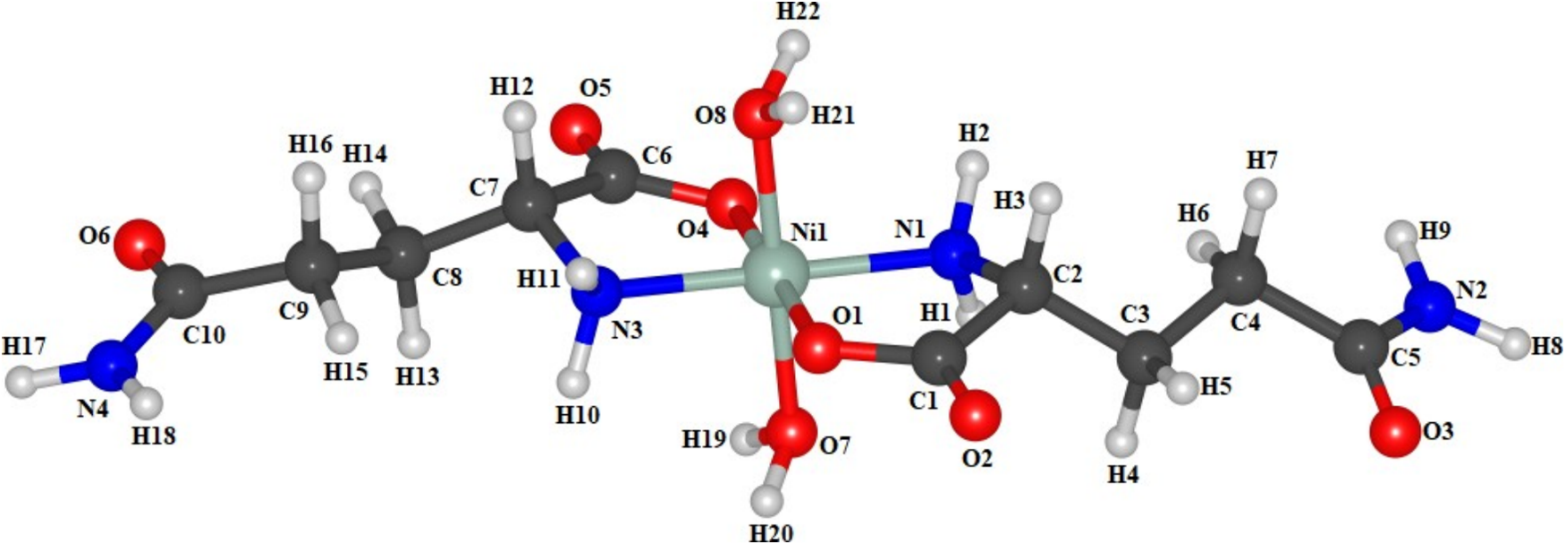
Optimized molecule of Ni(II)(l-glutaminato.H_2_O)_2_ calculated via DFT with the numbering of the atoms used in the VMARD.

Figure 11 shows the optimized molecule calculated via DFT with the number of atoms used in PED, respectively. The modes classified based on VMARD only considered movements with a percentage greater than 10%.

**Figure 12:**
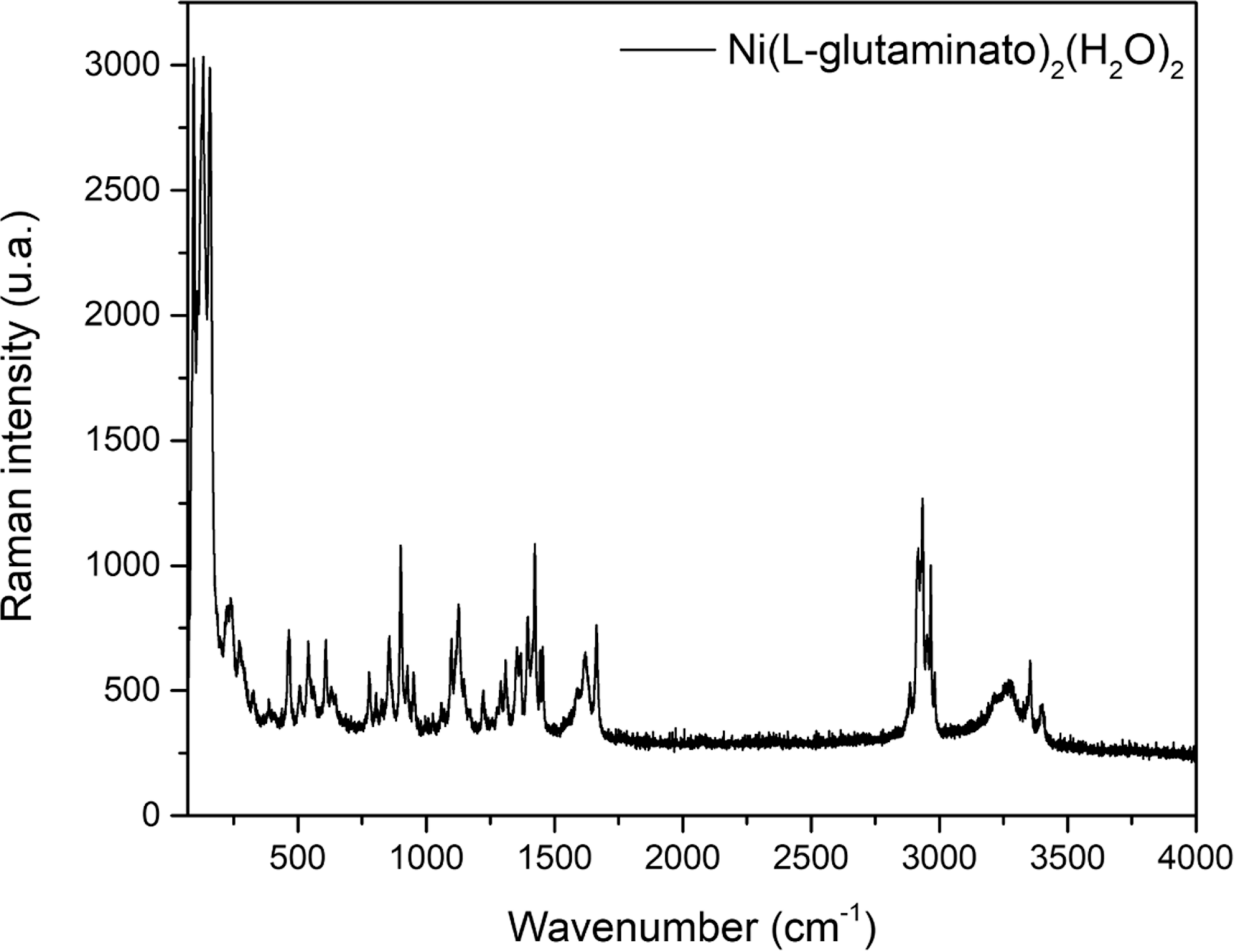
Experimental Raman spectrum at room temperature of the Ni(II)(l-glutaminato·H_2_O)_2_ crystal in the 70–4000 cm^–1^ range.

The Raman spectrum obtained experimentally is shown in Figure 12. Out of the 135A_g_ Raman-active modes predicted by group theory, approximately 48 were observed. This discrepancy between the expected and observed modes can be attributed to the presence of nearly degenerate vibrational modes, as there are numerous closely resembling atomic bonds with very similar vibrational energies (below the resolution limit of the spectrometer), as well as modes with low scattering cross sections (low intensity). Additionally, due to technical limitations, certain vibrational modes related to crystal lattice vibrations (<70 cm^–1^) could not be computed. All experimentally observed vibrational modes were accurately assigned based on the VMARD results, as detailed in Table 4.

**Table 4:** Calculated, Scaled, and Experimental Raman Modes (cm^–1^), with Assignments for Ni(II)(l-glutaminato.H_2_O)_2_ Crystal in the Region 70–4000 cm^–1^ at Room Temperature^a^

ω_calc_	ω_scal_	ω_exp_	assignments with VMARD (≥10%)
		93	
		107	
122	120	122	δ(C3C4C5) [13]
133	131	131	δ(C8C9C10) [10]
		156	
195	192		δ(O7Ni1O8) [12]
226	222	222	δ(O4Ni1O7) [10]
254	250	240	δ(C6C7C8) [10]
291	286	275	δ(O4Ni1O1) [11] + δ(N3Ni1O8) [10]
297	292	292	τ(H7C4C5O3) [14] + τ(H7C4C5N2) [14] + τ(C3C4C5N2) [10]
311	306		τ(C8C9C10N4) [16] + τ(O6C10N4H18) [10]
444	436	464	ν(Ni1O7) [19] + ν(Ni1O8) [18]
525	516	507	ν(Ni1O8) [17] + ν(Ni1O7) [16]
		541	
712	700		δ(Ni1O8H21) [11]
		778	
		804	
871	856	856	ν(C9C10) [10]
893	878		ν(C4C5) [14]
902	887	901	ν(C9C10) [11]
		926	
		950	
1067	1049	1060	ν(C8C9) [14] + ν(C10N4) [12]
1111	1092		ν(C10O6) [16] + ν(C10N4) [11]
1112	1093	1097	ν(C5O3) [18] + ν(C5N2) [12]
1150	1130	1125	ν(O4C6) [11]
		1149	
1208	1187		ν(O1C1) [10]
1230	1209	1223	ν(O4C6) [14]
1320	1298	1290	ν(C5N2) [12]
1325	1302		ν(C10N4) [17]
1332	1309	1309	ν(C5N2) [14]
1375	1352	1353	ν(C5N2) [12]
1378	1355		ν(C10N4) [13] + δ(C7C8H13) [12]
1386	1362	1367	δ(H3C2N1) [17]
1394	1370	1396	δ(H12C7N3) [19]
1432	1408		ν(C10N4) [19] + δ(C8C9H15) [12] + ν(C9C10) [10]
1440	1416	1414	ν(C5N2) [17] + δ(H6C4C5) [12]
1452	1427	1423	δ(H15C9H16) [29]
1453	1428		δ(H6C4H7) [24] + ν(C3C4) [11]
1475	1450	1444	δ(H4C3H5) [36] + δ(H6C4H7) [15]
1476	1451	1454	δ(H13C8H14) [33] + δ(H15C9H16) [14]
1595	1568		δ(H21O8H22) [40]
1606	1579		δ(H19O7H20) [44]
1616	1589	1587	δ(H17N4H18) [26] + ν(C10O6) [23] + ν(C10N4) [15] + δ(C10N4H18) [13]
1618	1590		δ(H8N2H9) [26] + ν(C5O3) [22] + ν(C5N2) [15] + δ(C5N2H9) [14]
1624	1596		δ(H10N3H11) [41] + δ(C7N3H10) [11]
1633	1605	1621	δ(H1N1H2) [36]
1671	1643		ν(C10O6) [59] + ν(C10N4) [12]
1673	1645	1663	ν(C5O3) [57] + ν(C5N2) [11]
3029	2893	2886	ν(C9H16) [51] + ν(C9H15) [19] + ν(C8H13) [19]
3030	2894		ν(C4H7) [51] + ν(C4H6) [23] + ν(C3H4) [17]
3045	2908	2912	ν(C8H13) [45] + ν(C8H14) [21] + ν(C9H16) [16]
3046	2909	2918	ν(C3H4) [43] + ν(C3H5) [19] + ν(C2H3) [14] + ν(C4H7) [12] + ν(C4H6) [10]
3069	2931	2927	ν(C2H3) [63] + ν(C3H5) [16] + ν(C3H4) [14]
3079	2940	2934	ν(C4H6) [52] + ν(C4H7) [24] + ν(C3H4) [14]
3085	2946		ν(C7H12) [46] + ν(C8H14) [22] + ν(C9H15) [22]
3088	2949	2950	ν(C9H15) [41] + ν(C7H12) [28] + ν(C9H16) [15] + ν(C8H14) [10]
3109	2969	2960	ν(C8H14) [35] + ν(C8H13) [18] + ν(C9H15) [15]
3110	2970	2982	ν(C3H5) [40] + ν(C3H4) [19] + ν(C4H6) [11]
		3161	
		3224	
3445	3290	3279	ν(N1H1) [30] + ν(N3H10) [26] + ν(N1H2) [23] + ν(N3H11) [20]
3451	3296		ν(N3H10) [30] + ν(N1H1) [26] + ν(N3H11) [23] + ν(N1H2) [20]
3520	3362	3354	ν(N1H2) [39] + ν(N1H1) [30] + ν(N3H11) [17] + ν(N3H10) [13]
3521	3363		ν(N3H11) [40] + ν(N3H10) [30] + ν(N1H2) [17] + ν(N1H1) [13]
3566	3406	3398	ν(N2H8) [52] + ν(N2H9) [46]
3569	3408		ν(N4H17) [53] + ν(N4H18) [44]
3656	3491		ν(O7H19) [31] + ν(O7H20) [30] + ν(O8H21) [19] + ν(O8H22) [17]
3667	3502		ν(O8H21) [32] + ν(O8H22) [29] + ν(O7H19) [18] + ν(O7H20) [18]
3691	3525		ν(N2H9) [53] + ν(N2H8) [46]
3695	3529		ν(N4H18) [55] + ν(N4H17) [45]
3736	3568		ν(O7H20) [49] + ν(O7H19) [47]
3740	3572		ν(O8H22) [50] + ν(O8H21) [46]

a Nomenclature: ν - stretching, δ - angular deformation, τ - twist, γ - out-of-plane deformation, ρ - rotation.

The crystal lattice vibration modes are found in the lower frequency region. The bands at 122, 131, and 240 cm^–1^ have been assigned to the angular deformations of the side chain of glutamine, specifically δ(CCC). According to the results of VMARD, the experimental modes at 222 and 275 cm^–1^ are attributed to deformations of the angles related to the metal–ligand bonds, specifically δ(ONiO) and δ(ONiO) + δ(NNiO), respectively. The vibrational mode at 292 cm^–1^ corresponds to the combination movements of the τ(HCCO_3_) + τ(HCCN) + τ(CCCN) skeleton. Finally, the bands at 464 and 507 cm^–1^ correspond to the combination of stretching movements of nickel with water molecules ν(NiO) + ν(NiO) and ν(NiO) + ν(NiO).

In the 700–1700 cm^–1^ region spectrum, the ν(CC) bond was observed at 856 and 901 cm^–1^ bands. The ν(OC) bond stretching is identified at 1125 and 1223 cm^–1^. The amide group’s ν(CN) stretching is located at 1290, 1309, and 1353 cm^–1^.

The 1060, 1097, and 1663 cm^–1^ bands were assigned via VMARD for CC, CN, and CO stretching-type movement combinations. The angular deformation δ(HCH) was found at 1423, 1444, and 1454 cm^–1^. The δ(HCN) deformation was identified in the 1367 and 1396 cm^–1^ bands, and the δ(HNH) deformation was found at 1621 cm^–1^. The bands at 1414 and 1587 cm^–1^ correspond to movement combinations of the type ν(CN) + δ(HCC) and δ(HNH) + ν(CO) + ν(CN) + δ(CNH), respectively. Finally, the movement combination of the amide group, ν(CO) + ν(CN), was identified at 1663 cm^–1^.

In the Raman region between 2800 and 3600 cm^–1^, characteristic stretching-type vibrations of the CH and CN units are expected, and as it is a hydrated crystal, characteristic stretching-type vibrations of the water molecules are also expected. In the bands at 2886, 2912, 2918, 2927, 2934, 2950, 2960, and 2982 cm^–1^, vibrations related to bond stretching ν(CH) are observed. NH stretches were found in the 3279, 3354, and 3398 cm^–1^ bands. Finally, the 3491, 3502, 3568, and 3572 cm^–1^ values (scaled wavenumbers) were designated as stretching of the OH bond referring to water molecules present in the material.

## 3 Conclusions

This study obtained a new nickel-containing l-glutamine complex by the solvent slow evaporation method, Ni(II)(l-glutaminato·H_2_O)_2_, which crystallized as a spherulite. The SEM images revealed the beautiful irregular honeycomb-like microstructure of the spherulite surface and its internal region formed by well-aligned microrods. The measurement of powder X-ray diffraction followed by searches on the Cambridge Crystallographic Data Center (CCDC) database confirmed that a new material was obtained. Several techniques were used to determine its crystalline structure. From the TGA technique, it was concluded that two water molecules were coordinated to the Ni atom, which corroborates with the Ni(II)(l-glutaminato·H_2_O)_2_ chemical formula. Based on this information, five conformations of probable spatial configurations for the molecule were assembled. The relaxed structures of the complex were used as the angular basis (0° and 0°) of the dihedrals ϕ and θ. For I and II conformations, the set of dihedrals (0°, 0°) presented the most stable structure possible, with 0.07 and 0.38 kcal mol^–1^ energy differences between the dihedrals (0°, 0°) and (120°, 0°), respectively. From the indexing of the peaks of the PXRD measurement in transmission geometry and with monochromatic radiation, it was seen that the new complex crystallized in a triclinic system with space group *P*1̅ and its lattice parameters were determined. The atomic positions were determined by using complexes I and II in the Simulated Annealing program. Due to lower *R*_wp_ values, the data of conformation II were used in Rietveld refinement to obtain the final crystal structure. Infrared and Raman measurements at room temperature were obtained, and vibrational modes were determined and classified by using DFT calculations. The main vibrations about functional groups of metal–ligand bonds were identified, confirming the formation of the complex.

## 4 Materials and Methods

### 4.1 Synthesis of Ni(II)(l-glutaminato·H_2_O)_2_ Complex

All of the reagents were used as received from commercial sources without further purification.

The Ni(C_5_H_9_N_2_O_3_)_2_(H_2_O)_2_ complex was obtained by the slow solvent evaporation method from an aqueous solution (20 mL) of l-glutamine and NiCl_2_·6H_2_O in a 2:1 molar ratio, with pH elevation to 8 by the addition of NaOH. The mixture was filtered and then placed in an oven at 30 °C. After a few days, small blue spherical polycrystals (spherulites) were obtained.

### 4.2 Powder X-ray Diffraction

The initial powder X-ray diffraction (PXRD) measurements were carried out on a Bruker AXS X-ray diffractometer, model D8 Advance, equipped with a LynxEye linear detector. The measurements were carried out at room temperature using Cu*K*α_1/2_ = 1.540596/1.54441 Å radiation, in Bragg–Brentano geometry, with a step size of 0.02°, count time of 0.5 s per step, and range of 5.00 ≤ 2θ ≤ 60.00°. Monochromatic PXRD data were collected at room temperature in transmission geometry (using a capillary stage) on a STADI-P powder X-ray diffractometer (Stoe, Darmstadt, Germany), operating at 40 kV and 40 mA, using Cu*K*α_1_ = 1.540596 Å radiation selected by a curved Ge(111) monochromator. A Mythen 1K detector recorded data from 5.00 to 60.78° (2θ), with a step size of 0.015° and an integration time of 100 s at each 1.05°. This monochromatic PXRD measurement was used to determine the structure of the new complex.

### 4.3 Scanning Electron Microscopy and Energy Dispersive Spectroscopy

The morphology and elemental analysis of the new nickel-containing l-glutamine spherulite complex was investigated by Scanning Electron Microscopy (SEM), using the Zeiss EVO 15 microscope, equipped with a Bruker XFlash 410-M EDS detector.

### 4.4 Thermogravimetric Analysis

Thermogravimetry data were collected by the simultaneous thermal analyzer STA 449 F3, model Netsch Jupiter, with a heating rate of 10 °C min^–1^, using nitrogen as purge gas (100 mL min^–1^) and open alumina crucibles.

### 4.5 Vibrational Analysis

The infrared (IR) spectrum was obtained using a Thermo Nicolet NEXUS 870 ESP FT-IR/NIR spectrometer, with KBr pellets, in the spectral region 400–4000 cm^–1^. Raman measurement was performed in the range 70–4000 cm^–1^ on a Horiba Raman spectrometer iHR550 coupled to a BX41 Olympus confocal microscope with an objective lens of 20.4 mm (20x) focal length. The sample was excited by a He–Ne laser (632.8 nm), collected through optical fibers, and detected by a charge-coupled device (CCD) thermoelectrically cooled to −70 °C. Laser power on the sample surface was <2 mW, with 2 cm^–1^ spectral resolution.

### 4.6 Computational Details

Quantum chemical calculations of Ni(C_5_H_9_N_2_O_3_)_2_(H_2_O)_2_ were performed using a DFT framework implemented by the ORCA code.^36^ The B3LYP^37,38^ exchange-correlation functional was used with triple-ζ basis sets, def2-TZVP for the Ni atom, and 6-311++G(d,p) for the remaining atoms. The initial geometries of the five proposed complexes were based on available hydrated crystalline structures of amino acid complexes with Ni^2+^ and Cu^2+^. Following this, we performed a conformational scan on the dihedrals of the glutamine lateral chains by scanning the potential energy surface (PES) at 60° intervals ranging from −180 to 180 deg. This process yielded 36 structures for each conformation, with the intent to identify the global minimum energy for each of the five proposed conformations. A conductor-like polarizable continuum model^39^ was adopted to simulate the aqueous environment. Theoretical Raman- and IR-active frequencies were scaled using a scaling factor of 0.958 to wavenumbers calculated above 1700 cm^–1^, and 0.983 was applied to wavenumbers calculated below 1700 cm^–1^. The VibAnalysis version 1.2.2 program^40^ was used to analyze vibrational modes in terms of the automatic determination of vibrational mode relevance (VMARD). Only contributions with a percentage greater than 10% were taken into consideration.

### 4.7 Structure Determination

All stages of the structure determination process (indexing, Pawley refinement, simulated annealing, and Rietveld refinement) were carried out using the TOPAS-academic V7 program.^41,42^ For the indexing procedure,^43^ the first 23 peaks of the monochromatic PXRD measurement were selected. After analyzing the systematic absences, the crystal system, space group, and lattice parameters were identified and confirmed by Pawley fit.^44^ The values obtained by Pawley fit were used as input for the structure determination procedure, which employed a simulated annealing procedure implemented by TOPAS-Academic v7. Five possible 3D models of the Ni(II)(l-glutaminato·H_2_O)_2_ molecule were created and optimized by DFT calculations. The rigid body structure was built for the five optimized models, which were then used in the simulated annealing procedure.^20,45^ The full range of possible values of molecular positions and orientations and any flexible torsion angles (three describing the positional coordinates, four of which three are independent, describing the molecular orientation, and two flexible torsion angles) were allowed to vary during the simulated annealing process. Fifteen runs (for a total of 3 × 10^8^ movements) were globally optimized. The best result was considered in the final Rietveld refinement^46^ of the structure using the program TOPAS-Academic V7.

## Supporting Information Available

Additional figures depicting additional MEV images, Pawley fit, PES graphics, and crystallographic data like bond lengths and angles (PDF)
