# Supplementary material for: Spherulite-like Ni(II)(l-glutaminato·H2O)2 Complex: Morphological Microstructure, DFT-Assisted Crystal Structure Determination, and Vibrational Analysis
Source: ACS Omega. 2025 Mar 2;10(9):9503–13. doi: 10.1021/acsomega.4c10609 (PMC11904666; doi:10.1021/acsomega.4c10609)
Supplement: Supplementary file 1 — ao4c10609_si_001.pdf [file ao4c10609_si_001.pdf]

The spherulite-like Ni(II)(L-glutaminato·$H_2O)_2$ complex: morphological microstructure, DFT-assisted crystal structure determination, and vibrational analysis

Wesley K. C. Oliveira[1], Raisa M. C. S. Diniz[3], Jéssica A. O. Rodrigues[2], Francisco F. de Sousa[2,8], Clenilton C. dos Santos[1], Francisco S. M. Sinfrônio[4,5], Fabio F. Ferreira[6], José G. da Silva Filho[7], Alan S. de Menezes[1,2]*

1. Department of Physics, CCET, Federal University of Maranhão - UFMA, 65080-805, São Luís, MA, Brazil
2. Center for Social Sciences, Health, and Technology, Federal University of Maranhão - UFMA, 65900-410, Imperatriz, MA, Brazil
3. Department of Mathematics and Informatics, State University of Maranhão - UEMA, 65055-310, São Luís, MA, Brazil
4. Department of Electrical Engineering, Federal University of Maranhão, CCET, 65080-805, São Luís, MA, Brazil.
5. Institute of Chemistry, Federal University of Rio Grande do Norte - UFRN, 59078-970, Natal, RN, Brazil.
6. Center for Natural and Human Sciences, Federal University of ABC (UFABC), 09210-580, Santo André, SP, Brazil.
7. Faculdade de Educação, Ciências e Letras do Sertão Central, Universidade Estadual do Ceará - UECE, 63902-098, Quixadá, CE, Brazil.
8. Institute of Exact and Natural Sciences, Federal University of Para - UFPA, 66075-110, Belém, PA, Brazil

[*]Corresponding author
E-mail: alan.menezes@ufma.br (Alan S. de Menezes)

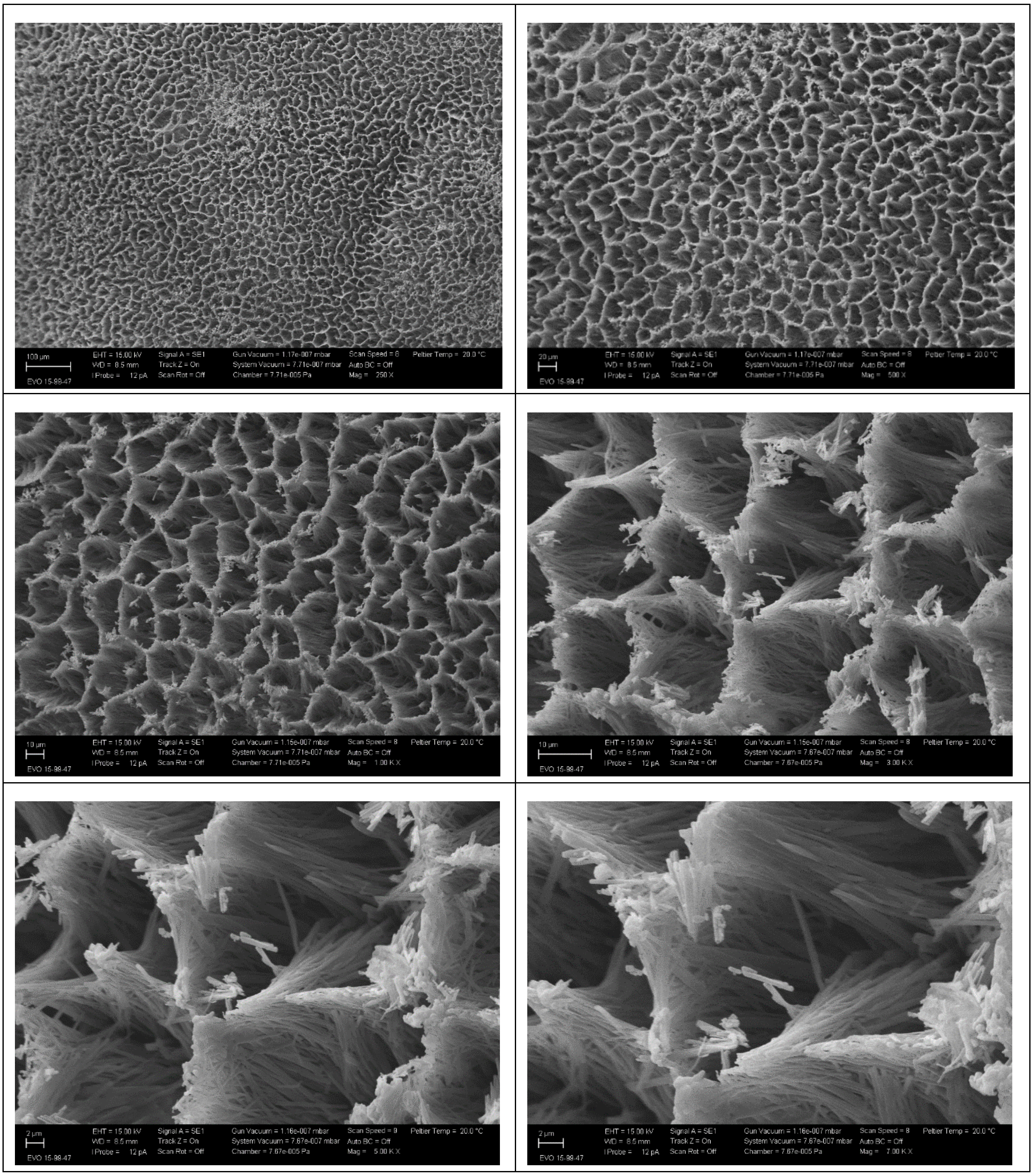

Figure S01. SEM images of the new Ni(II)(L-glutaminato·H_2O)$_2$ spherulite top surface.

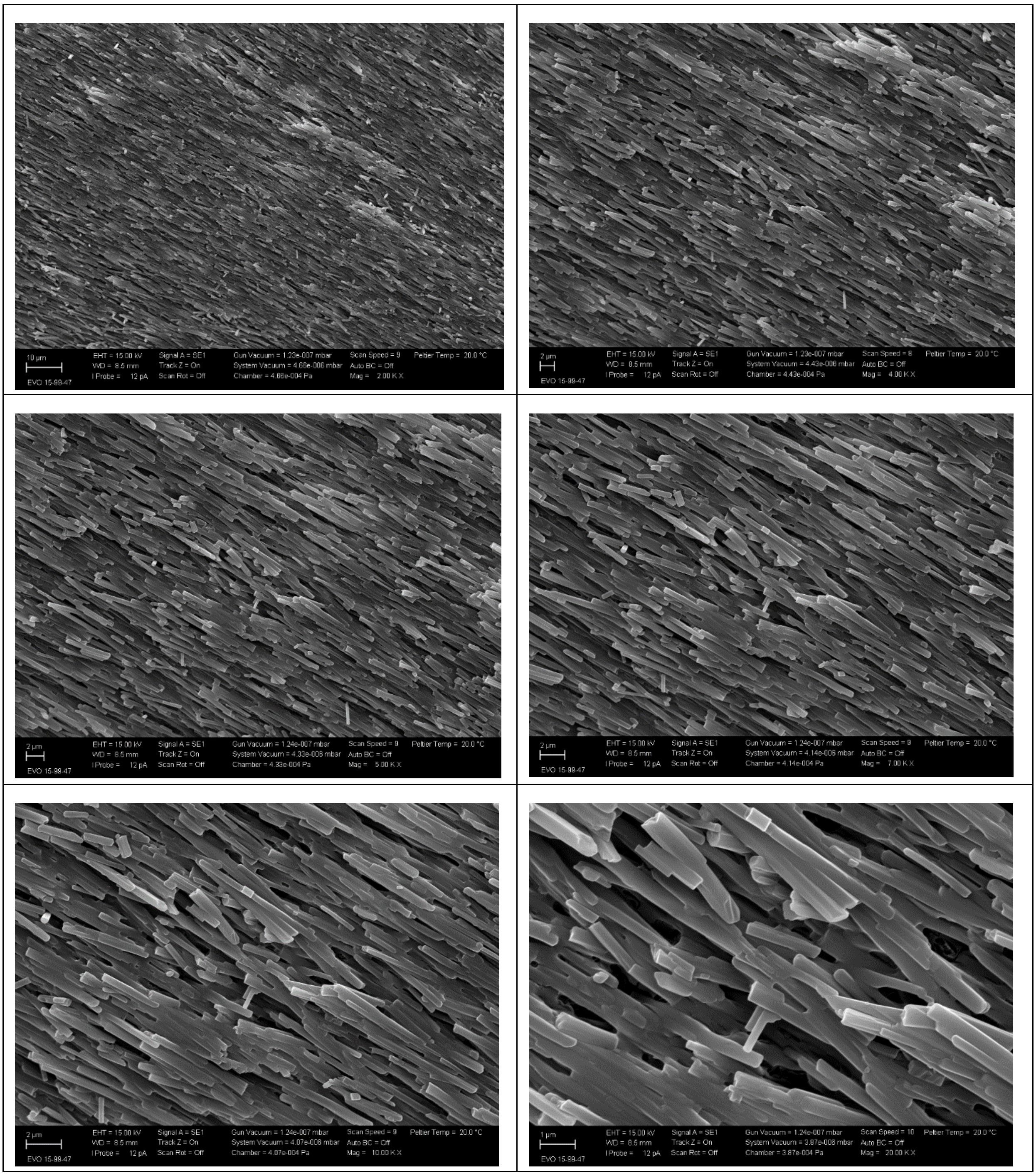

Figure S02. SEM images of the new Ni(II)(L-glutaminato·H_2O)$_2$ spherulite internal region.

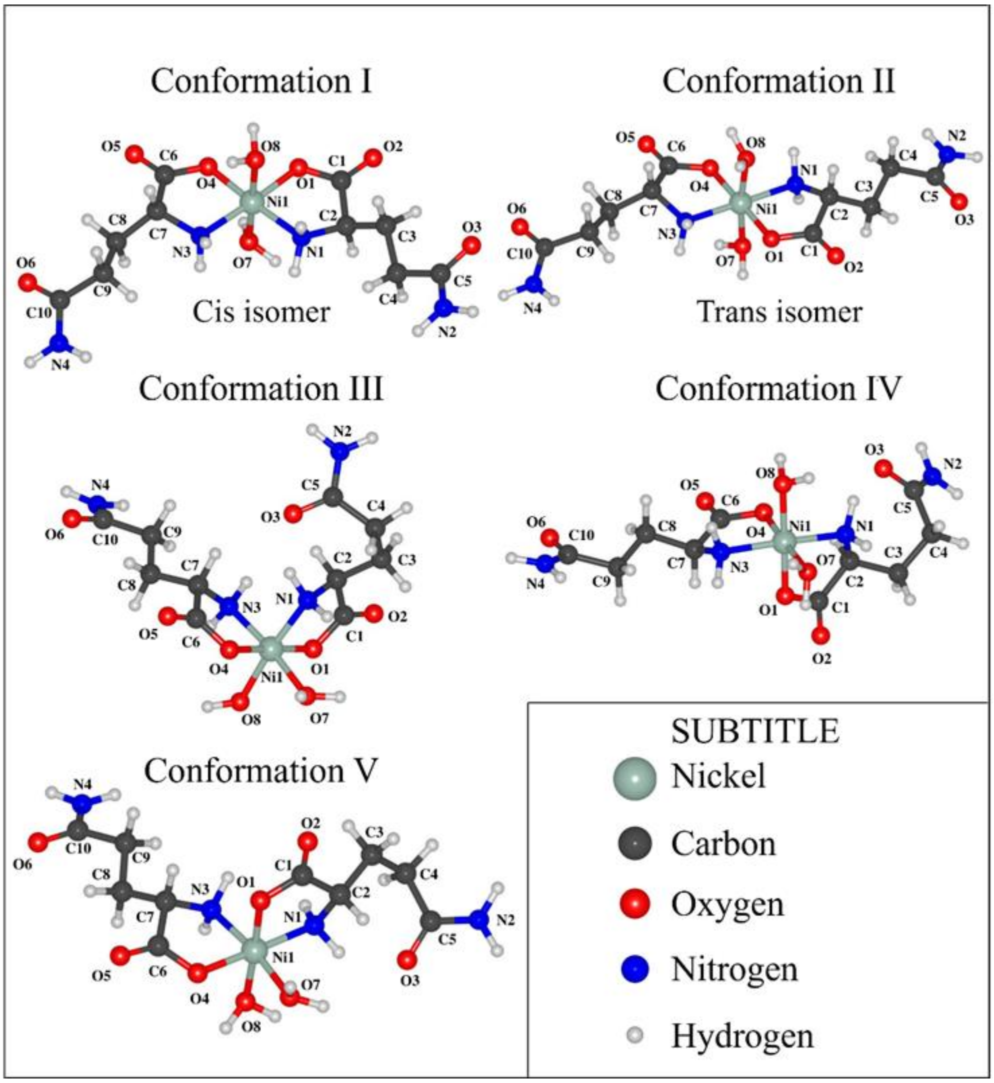

Figure S03. Three-dimensional structural representation of the five conformations studied of the Ni(II)(L-glutaminato·H_2O)$_2$ complex.

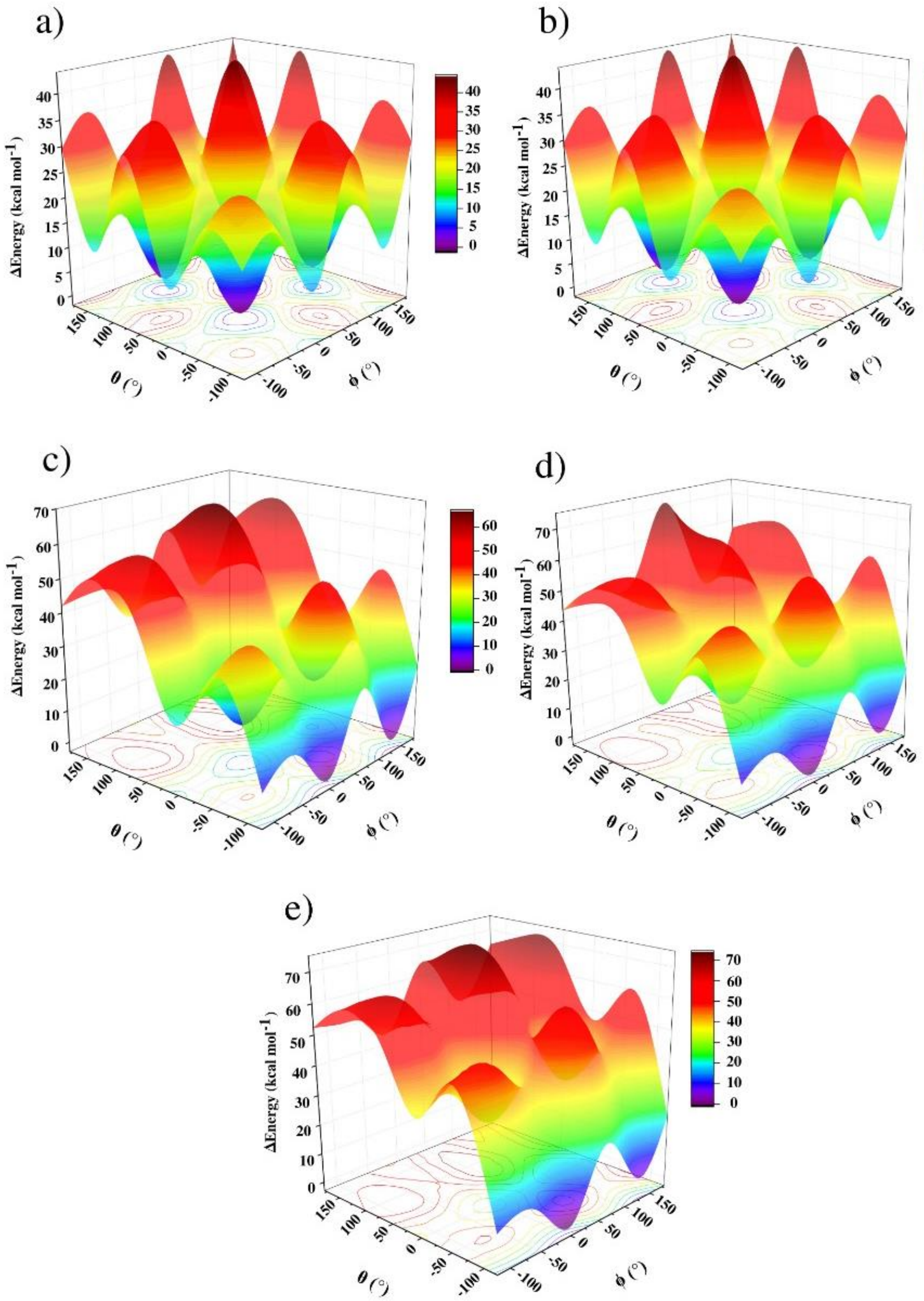

Figure S04. Potential energy surfaces for the five complex conformations. a) conformation I, b) conformation II, c) conformation III, d) conformation IV, and e) conformation V.

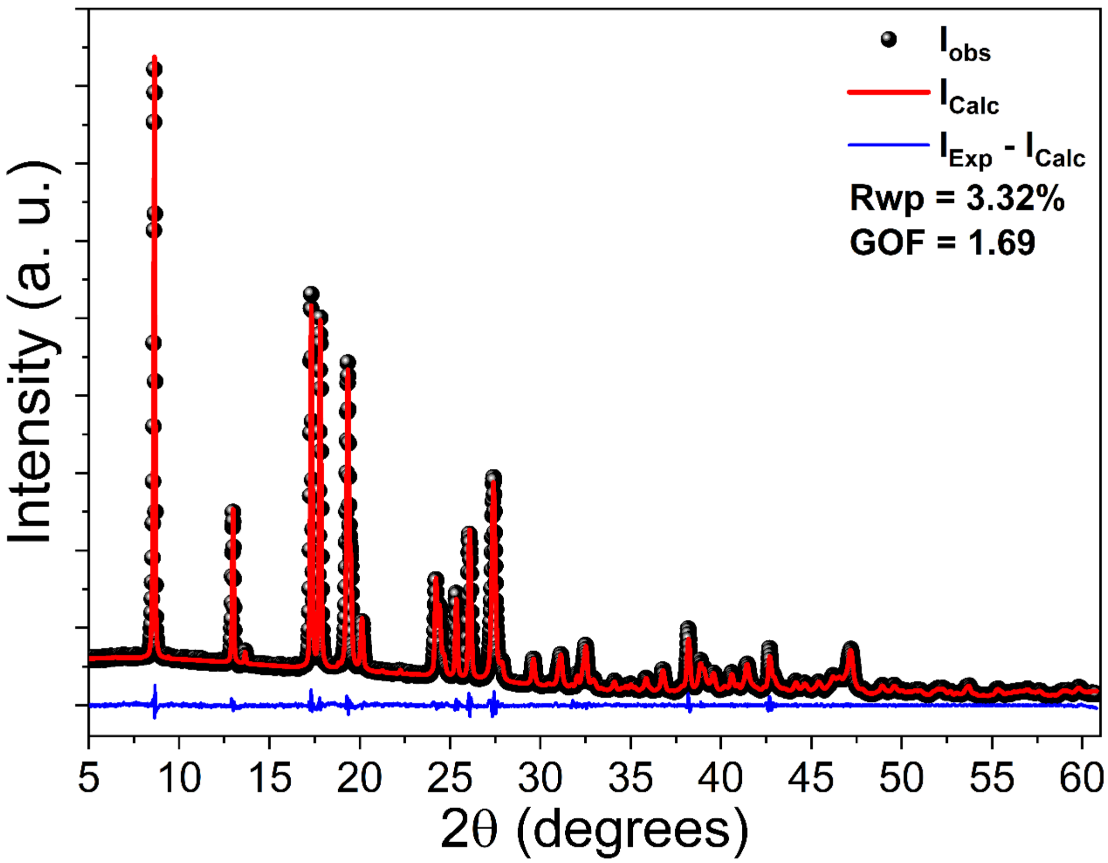

Figure S05. Pawley refinement graph.

Table S01 – Bond lengths (Å), bond angles (°) and dihedral angles (°) of five Ni(II)(L-glutaminato·H_2O)$_2$ molecule conformations obtained by DFT calculations with B3LYP/6-311++G(d,p) and def2-TZVP.

B3LYP/6-311++G(d,p) and def2-TZVP					
Coordinates	Conf-I	Conf-II	Conf-III	Conf-IV	Conf-V
Ni-H_2O (H-O7-H)	1.900	1.904	1.962	1.961	1.960
Ni-H_2O (H-O8-H)	1.900	1.897	1.976	1.963	1.965
Ni-NH_2 (H-N1-H)	1.964	1.952	1.912	1.937	1.945
Ni-NH_2 (H-N3-H)	1.965	1.959	1.914	1.953	1.917
Ni-COO (C1-O1-O2)	2.726	2.717	2.732	2.700	2.714
Ni-COO (C6-O4-O5)	2.734	2.730	2.724	2.699	2.722
C-C	1.524	1.528	1.529	1.527	1.527
C-N	1.415	1.417	1.414	1.413	1.415
C-O	1.253	1.256	1.258	1.260	1.258
COO-Ni-NH_2	60.16°	60.50°	60.31°	60.16°	60.18°

Table S02 – Atomic positions and sites of Ni(II)(L-glutaminato·$H_2O)_2$ atoms.

Atom	Site	x	y	z
Ni1	2i	0.2375(10)	0.4990(4)	0.2498(3)
O1	2i	-0.0739(10)	0.5022(4)	0.1478(3)
O2	2i	-0.2348(10)	0.6066(4)	0.0442(3)
03	2i	0.1407(10)	1.0617(4)	0.1350(3)
O4	2i	0.5607(10)	0.4949(4)	0.3443(3)
O5	2i	0.7115(10)	0.3902(4)	0.4445(3)
O6	2i	-0.0091(10)	-0.0775(4)	0.3659(3)
O7	2i	0.4554(10)	0.3839(4)	0.1518(3)
O8	2i	0.0348(10)	0.6201(4)	0.3509(3)
N1	2i	0.3680(10)	0.6559(4)	0.2014(3)
N2	2i	0.5812(10)	1.1018(4)	0.1374(3)
N3	2i	0.0996(10)	0.3359(4)	0.2896(3)
N4	2i	0.4054(10)	-0.1419(4)	0.3915(3)
C1	2i	-0.0679(10)	0.5912(4)	0.1049(3)
C2	2i	0.1854(10)	0.6810(4)	0.1237(3)
C3	2i	0.1459(10)	0.8181(4)	0.1155(3)
C4	2i	0.4055(10)	0.8855(4)	0.1098(3)
C5	2i	0.3597(10)	1.0242(4)	0.1168(3)
C6	2i	0.5438(10)	0.4058(4)	0.3860(3)
C7	2i	0.2985(10)	0.3127(4)	0.3644(3)
C8	2i	0.2754(10)	0.2006(4)	0.4141(3)
C9	2i	0.3427(10)	0.0699(4)	0.3554(3)
H1	2i	0.3873(10)	0.7249(4)	0.2503(3)
H2	2i	0.5186(10)	0.6391(4)	0.1799(3)
H3	2i	0.2716(10)	0.6308(4)	0.0730(3)
H4	2i	0.0278(10)	0.8159(4)	0.0575(3)
H5	2i	0.0718(10)	0.8658(4)	0.1719(3)
H6	2i	0.4683(10)	0.8467(4)	0.0487(3)
H7	2i	0.5327(10)	0.8778(4)	0.1629(3)
H8	2i	0.7306(10)	1.0868(4)	0.1650(3)
H9	2i	0.5336(10)	1.1708(4)	0.1160(3)
H10	2i	-0.0504(10)	0.3508(4)	0.3130(3)
H11	2i	0.0756(10)	0.2718(4)	0.2370(3)
H12	2i	0.2176(10)	0.3698(4)	0.4150(3)
H13	2i	0.3696(10)	0.2208(4)	0.4740(3)
H14	2i	0.0786(10)	0.1958(4)	0.4240(3)
H15	2i	0.2306(10)	0.0548(4)	0.2900(3)
H16	2i	0.5176(10)	0.0768(4)	0.3440(3)
H17	2i	0.3249(10)	-0.2050(4)	0.4078(3)
H18	2i	0.5696(10)	-0.1393(4)	0.3818(3)
H19	2i	0.3836(10)	0.3598(4)	0.0820(3)
H20	2i	0.6306(10)	0.4298(4)	0.1580(3)
H21	2i	-0.1353(10)	0.5755(4)	0.3541(3)
H22	2i	0.1411(10)	0.6408(4)	0.4163(3)

Table S03 – Lengths of the bonds of the molecule.

Bond lengths (Å)			
Bonds	Experimental	Calculated	Difference
C1-C2	1.549(7)	1.534	0.015
C2-C3	1.504(6)	1.528	0.024
C3-C4	1.516(7)	1.529	0.013
C4-C5	1.476(6)	1.523	0.047
C1-O1	1.245(7)	1.327	0.082
C1-O2	1.187(7)	1.208	0.021
C2-N1	1.444(6)	1.483	0.039
C5-O3	1.235(7)	1.233	0.002
C5-N2	1.346(7)	1.348	0.002
Ni1-O1	2.036(6)	1.862	0.174
Ni1-N1	2.075(7)	1.952	0.123
Ni1-O7	2.066(6)	1.904	0.162
Ni1-O8	2.072(6)	1.897	0.175
C6-C7	1.533(7)	1.532	0.001
C7-C8	1.534(7)	1.527	0.007
C8-C9	1.501(6)	1.530	0.029
C9-C10	1.501(7)	1.523	0.022
C6-O4	1.239(7)	1.328	0.089
C6-O5	1.170(7)	1.208	0.038
C7-N3	1.457(7)	1.488	0.031
C10-O6	1.250(7)	1.233	0.017
C10-N4	1.388(7)	1.348	0.040
Ni1-O4	2.018(7)	1.861	0.157
Ni1-N3	2.080(7)	1.959	0.121
N1-H1	0.870(6)	1.023	0.153
N1-H2	0.870(7)	1.021	0.151
C2-H3	0.945(6)	1.093	0.148
C3-H4	0.960(6)	1.095	0.135
C3-H5	0.960(6)	1.091	0.131
C4-H6	0.960(6)	1.093	0.133
C4-H7	0.960(6)	1.097	0.137
N2-H8	0.857(7)	1.010	0.153
N2-H9	0.888(6)	1.009	0.121
N3-H10	0.876(7)	1.023	0.147
N3-H11	0.871(6)	1.021	0.150
C7-H12	0.960(6)	1.094	0.134
C8-H13	0.902(6)	1.095	0.193
C8-H14	1.037(7)	1.091	0.054
C9-H15	1.009(6)	1.092	0.083
C9-H16	0.934(7)	1.096	0.162

N4-H17	0.870(6)	1.011	0.141
N4-H18	0.870(7)	1.009	0.139
O7-H19	0.985(6)	0.976	0.009
O7-H20	0.996(7)	0.976	0.020
O8-H21	0.993(7)	0.975	0.018
O8-H22	0.992(6)	0.975	0.017

Table S04 – Bond angles (˚).

Bond angles (˚)			
Bonds	Experimental	Calculated	Difference
C1-C2-C3	115.5(4)	112.4	3.10
C2-C3-C4	110.5(4)	112.5	2.00
C3-C4-C5	107.7(4)	111.8	4.10
C4-C5-O3	117.2(4)	121.9	4.70
C4-C5-N2	113.9(4)	115.5	1.60
O3-C5-N2	124.4(5)	122.6	1.80
O1-C1-O2	124.7(5)	120.8	3.90
O1-C1-C2	118.4(4)	115.3	3.10
O2-C1-C2	116.6(4)	123.8	7.20
N1-C2-C1	111.2(4)	108.5	2.70
N1-C2-C3	120.6(4)	112.5	8.10
Ni1-O1-C1	116.7(4)	115.9	0.80
Ni1-N1-C2	111.9(3)	109.2	2.70
O7-Ni1-O8	177.3(3)	172.8	4.50
O7-Ni1-O1	94.8(3)	88.1	6.70
O7-Ni1-N1	87.2(2)	86.8	0.40
O8-Ni1-O1	87.1(3)	89.3	2.20
O8-Ni1-N1	91.2(3)	86.3	4.90
C6-C7-C8	122.0(4)	112.0	10.00
C7-C8-C9	115.2(4)	112.9	2.30
C8-C9-C10	115.7(4)	111.3	4.40
C9-C10-O6	121.2(4)	121.6	0.40
C9-C10-N4	115.6(4)	115.8	0.20
O6-C10-N4	118.4(4)	122.6	4.20
O4-C6-O5	121.7(5)	120.8	0.90
O4-C6-C7	121.2(4)	115.2	6.00
O5-C6-C7	117.1(4)	123.9	6.80
N3-C7-C6	114.3(4)	108.4	5.90
N3-C7-C8	123.6(4)	112.7	10.90
Ni1-O4-C6	112.4(4)	116.8	4.40

Ni1-N3-C7	106.4(3)	109.8	3.40
O7-Ni1-O4	82.1(2)	90.6	8.50
O7-Ni1-N3	90.7(3)	93.4	2.70
O8-Ni1-O4	95.9(3)	92.0	3.90
O8-Ni1-N3	91.0(3)	93.5	2.50
O1-Ni1-O4	176.4(3)	178.7	2.30
O1-Ni1-N1	81.3(2)	86.4	5.10
O1-Ni1-N3	96.2(3)	94.2	2.00
O4-Ni1-N1	96.7(3)	93.5	3.20
O4-Ni1-N3	85.7(2)	85.8	0.10
N1-Ni1-N3	176.6(3)	179.3	2.70
Ni1-N1-H1	108.8(5)	105.28	3.52
Ni1-N1-H2	108.8(5)	107.88	0.92
Ni1-N3-H10	110.1(5)	106.87	3.23
Ni1-N3-H11	107.5(5)	108.96	1.46
Ni1-O7-H19	118.1(5)	112.97	5.13
Ni1-O7-H20	107.8(4)	114.83	7.03
Ni1-O8-H21	109.5(4)	115.98	6.48
Ni1-O8-H22	109.5(5)	116.26	6.76
C1-C2-H3	97.1(5)	105.81	8.71
C2-C3-H4	109.2(5)	109.26	0.06
C2-C3-H5	109.2(5)	106.69	2.51
C2-N1-H1	108.9(5)	112.36	3.46
C2-N1-H2	108.9(5)	112.98	4.08
C3-C2-H3	113.4(5)	110.38	3.02
C3-C4-H6	109.9(5)	111.52	1.62
C3-C4-H7	109.9(5)	109.69	0.21
C4-C3-H4	109.2(5)	110.82	1.62
C4-C3-H5	109.2(5)	109.96	0.76
C5-C4-H6	109.9(5)	109.91	0.01
C5-C4-H7	109.9(5)	106.73	3.17
C5-N2-H8	127.3(6)	119.70	7.60
C5-N2-H9	103.4(6)	121.86	18.46
C6-C7-H12	91.0(5)	106.10	15.1
C7-C8-H13	110.7(5)	109.08	1.62
C7-C8-H14	104.0(5)	106.70	2.70
C7-N3-H10	110.4(5)	111.47	1.07
C7-N3-H11	113.2(5)	112.44	0.76
C8-C7-H12	90.9(4)	110.55	19.65

C8-C9-H15	105.7(5)	111.45	5.75
C8-C9-H16	107.7(5)	109.67	1.97
C9-C8-H13	110.6(5)	110.66	0.06
C9-C8-H14	108.6(5)	109.90	1.30
C10-C9-H15	96.3(5)	110.22	13.92
C10-C9-H16	122.2(5)	106.82	15.38
C10-N4-H17	109.5(6)	119.67	10.17
C10-N4-H18	125.2(5)	121.92	3.28
N1-C2-H3	94.4(5)	106.95	12.55
N3-C7-H12	91.0(5)	106.87	15.87
H1-N1-H2	109.5(7)	108.78	0.72
H4-C3-H5	109.4(6)	107.40	2.00
H6-C4-H7	109.5(6)	106.91	2.59
H8-N2-H9	129.4(7)	118.37	11.03
H10-N3-H11	109.2(7)	107.09	2.11
H13-C8-H14	107.3(6)	107.35	0.05
H15-C9-H16	107.1(6)	107.17	0.07
H17-N4-H18	125.3(7)	118.38	6.92
H19-O7-H20	107.9(6)	108.19	0.29
H21-O8-H22	109.5(5)	108.69	0.81

Table S05 – Hydrogen bond lengths and angles.

Donor – H ···Acceptor	D–H (Å)	H···A (Å)	D···A(Å)	D - H···A (°)
O7 – H19 ··· O2	0.985(6)	1.984(6)	2.909(6)	155.4(5)
O7 – H20 ··· O1	0.996(7)	1.720(7)	2.707(7)	170.5(5)
O8 – H21 ··· O4	0.993(7)	1.734(7)	2.723(7)	173.0(5)
O8 – H22 ··· O5	0.992(6)	2.140(6)	3.073(6)	156.0(5)
N1 – H1 ··· N4	0.870(6)	2.139(6)	2.987(6)	164.7(5)
N1 – H2 ··· O1	0.870(7)	2.595(7)	3.400(7)	154.4(5)
N1 – H2 ··· O2	0.870(7)	2.387(6)	3.154(7)	147.3(5)
N3 – H10 ··· O4	0.876(7)	2.557(7)	3.343(7)	149.8(5)
N3 – H10 ··· O5	0.876(7)	2.308(6)	3.096(7)	149.8(5)
N3 – H11 ··· O3	0.871(6)	2.393(6)	3.210(6)	156.2(5)
N2 – H8 ··· O3	0.857(7)	2.205(7)	2.915(7)	140.3(5)
N2 – H9 ··· O7	0.888(6)	2.247(6)	3.038(6)	148.2(5)
N4 – H17 ···O8	0.870(6)	2.269(7)	3.017(7)	144.0(5)

N4 – H18 ⋯ O6	0.870(7)	2.295(7)	3.146(7)	165.7(5)
C8 – H13 ⋯ N4	0.902(6)	2.452(6)	3.249(6)	147.4(5)
C9 – H15 ⋯ O3	1.009(6)	2.217(6)	3.162(6)	155.3(5)
